# The mRNA-Based Innovative Strategy: Progress and Challenges

**DOI:** 10.1007/s40820-025-01906-x

**Published:** 2026-01-15

**Authors:** Huayuan Zhou, Dali Wei, Zhejie Chen, Hao Chen, Chuhuang Dong, Wei Yao, Jiawen Wang, Xueliang Liu, Yuqing Li, Yu Yang, Weihong Tan

**Affiliations:** 1 Institute of Molecular Medicine (IMM), School of Medicine, Renji Hospital, Shanghai Jiao Tong University, Shanghai, 200240 People’s Republic of China; 2 School of Life Sciences, Shanghai University, Shanghai, 200444 People’s Republic of China; 3 Institute of Functional Nano & Soft Materials (FUNSOM), Soochow University, Suzhou, 215123 People’s Republic of China

**Keywords:** mRNA structure optimization, mRNA delivery system, mRNA therapeutics

## Abstract

As the central template for protein expression, messenger ribonucleic acid (mRNA) holds immense potential for novel therapeutic strategies. Over the past few decades, mRNA-based therapeutics have demonstrated remarkable efficacy in a range of applications, including epidemic vaccine, cancer vaccine, protein replacement therapy, cytokine therapy, cell therapy and gene editing. Due to the inherent instability of mRNA, the rational design of mRNA structure is the prerequisite for therapeutic utility while effective delivery systems are also essential for in vivo applications. This review focuses on the optimization of mRNA structure and highlights key delivery strategies. It also provides a comprehensive overview of the major applications of mRNA-based strategies. In addition, it highlights the persistent challenges in mRNA therapeutics, particularly in terms of stability, immunogenicity, delivery efficiency and safety. By examining recent advances in mRNA design, delivery and application, this review aims to support ongoing research and development in the field of mRNA-based therapeutics.

## Highlights

- Messenger ribonucleic acid (mRNA) structural optimization and delivery systems were comprehensively summarized.
- Current mRNA applications were thoroughly introduced.
- The challenges and future prospects of mRNA-based therapeutics were critically analyzed and discussed.

## Graphical abstract

**Figure:**
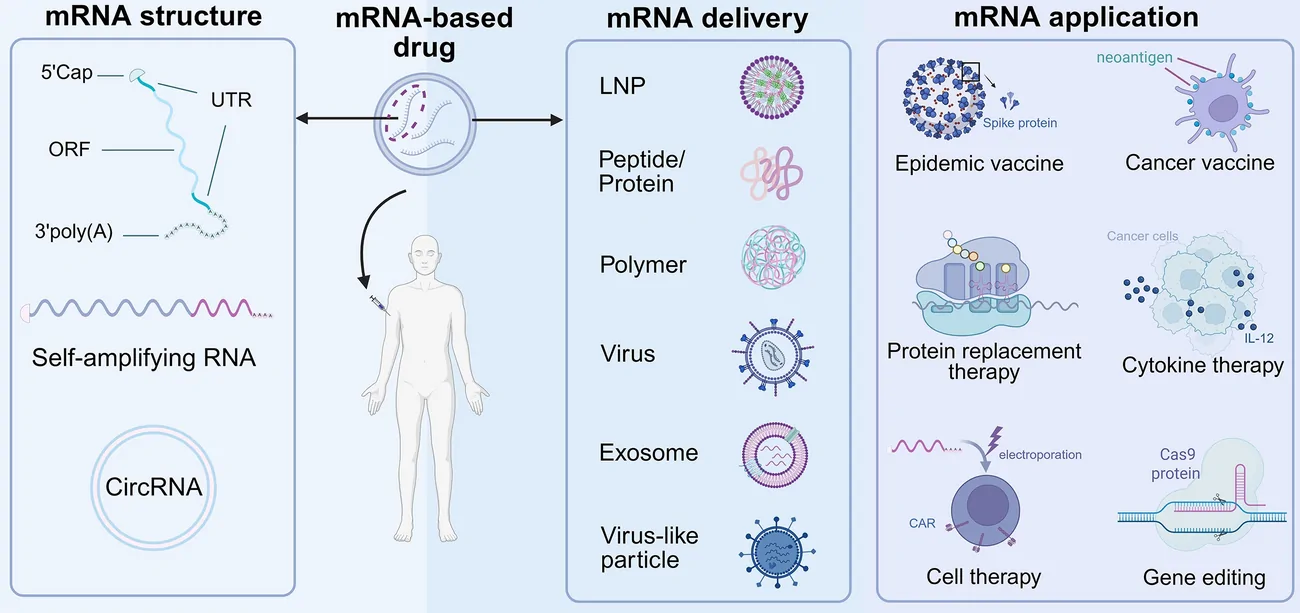

## Introduction

**Fig. 1:**
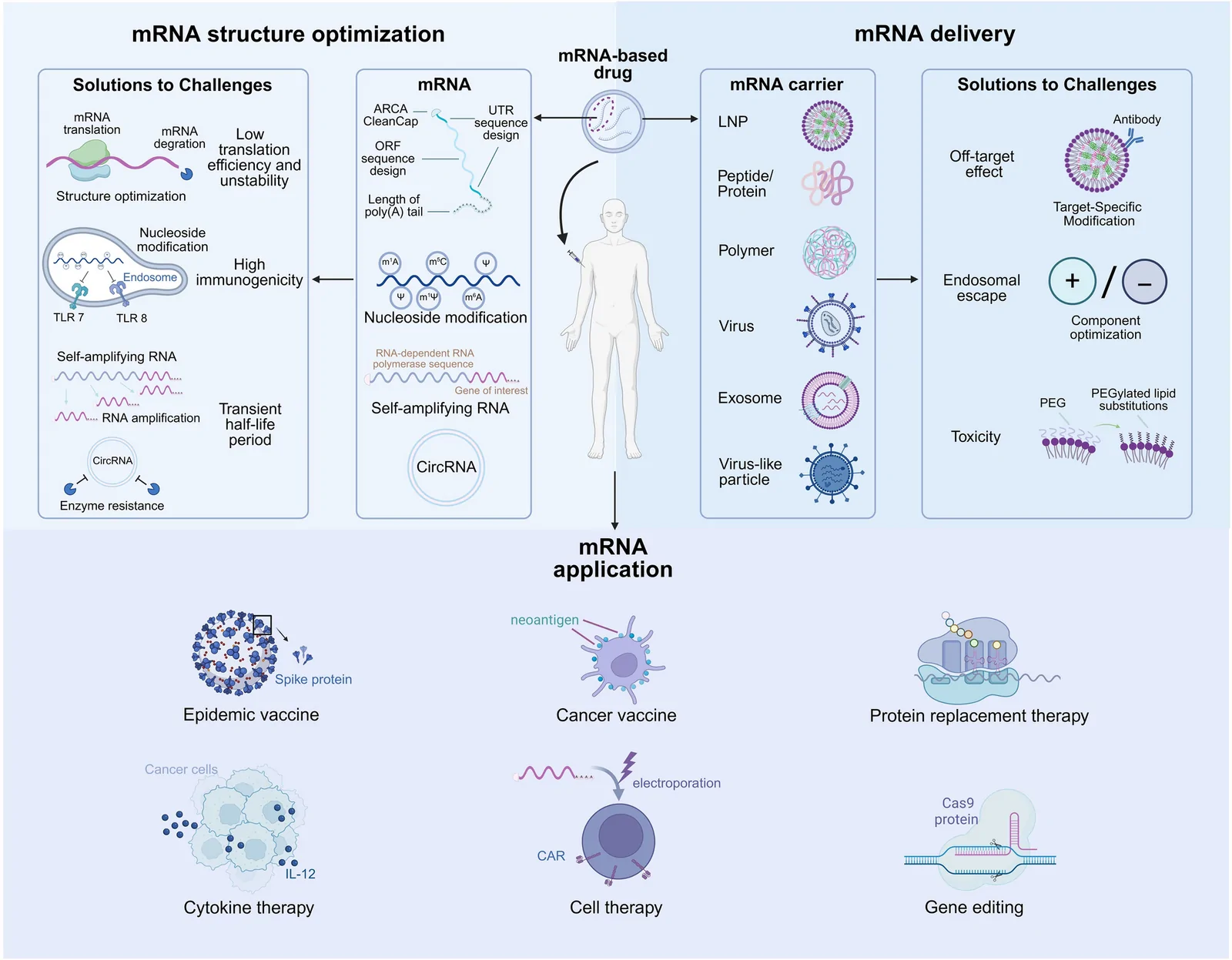
Schematic representation of mRNA structure optimization, delivery strategies and clinical/preclinical applications. Created with BioRender.com.

**Fig. 2:**
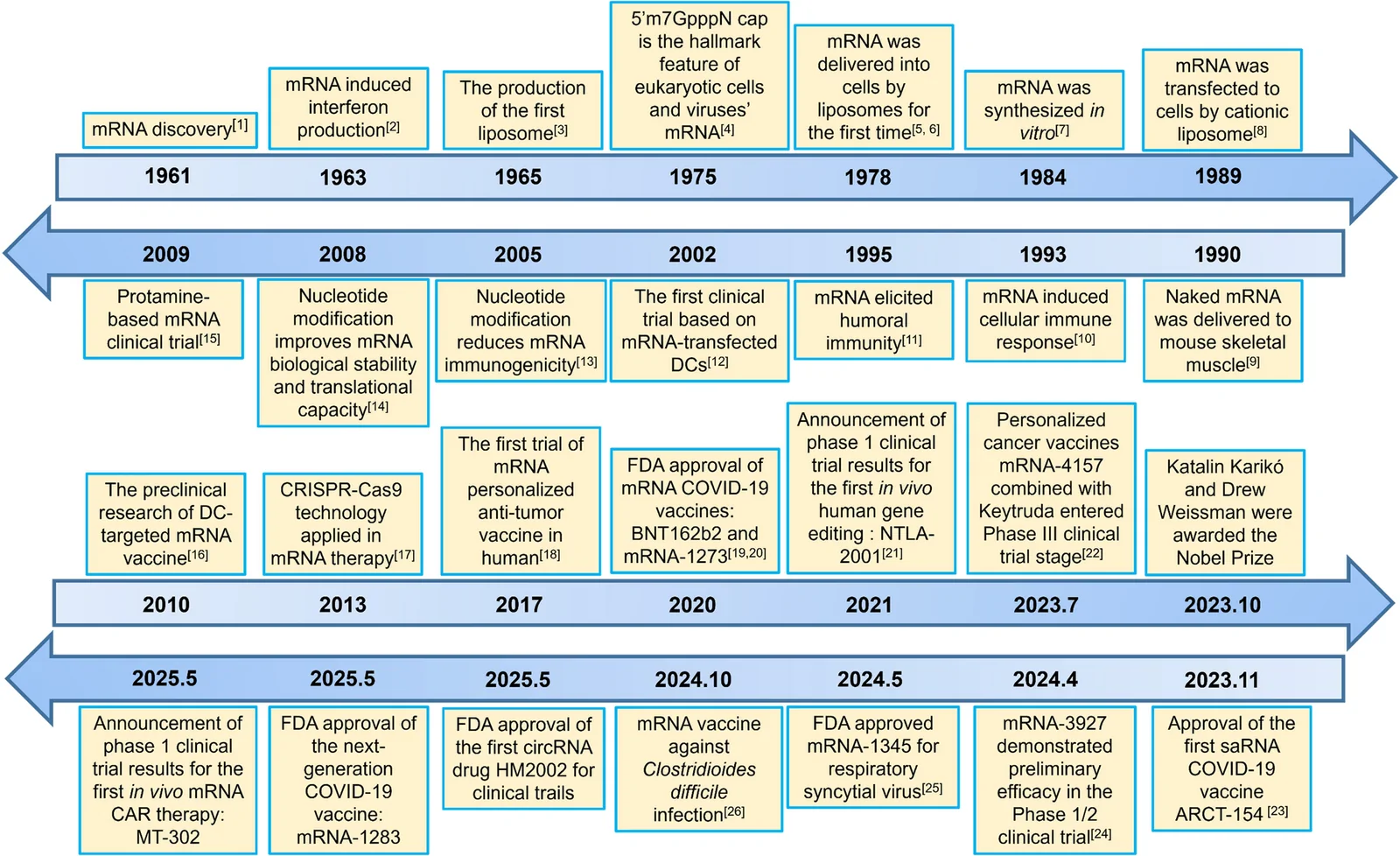
Development history of mRNA-based therapeutics.

Messenger ribonucleic acid (mRNA), first discovered in the 1960s, is characterized as an unstable intermediate carrying information from genes to ribosomes for protein synthesis [1]. Although mRNA constitutes only 2%–5% of the total RNA in cells, it can directly mediate the production of bioactive proteins, which enables its development as an effective therapeutic through rational mRNA design and delivery (Fig. 1). Therefore, researchers have long pursued the artificial synthesis of mRNA for biomedical applications [1–26] (Fig. 2). In 1978 and 1990, the first intracellular delivery of mRNA and its delivery in murine models were achieved, respectively [5, 6, 9]. However, in the subsequent decades, the immunostimulatory property of mRNA made it prone to recognition and inactivation by the immune system, posing a formidable obstacle to further research. The year 2005 marked a pivotal breakthrough in mRNA therapeutics when Katalin Karikó and Drew Weissman made the seminal discovery that nucleoside modification critically modulates Toll-like receptor (TLR)-mediated recognition of exogenous RNA [13]. This foundational work established the biochemical basis for all clinically viable mRNA platforms, which earned them the 2023 Nobel Prize in Physiology or Medicine.

The efficacy of mRNA-based therapeutics is determined by the stability and translational efficiency of mRNA, which are closely associated with its structural features and delivery systems. Building upon mRNA structure optimization and delivery vector improvement, mRNA vaccines have demonstrated significant clinical efficacy during the COVID-19 pandemic, highlighting hallmark advantages of this platform including expedited manufacturing timelines, superior protective efficacy and eliminated genomic integration risks [20, 27]. The remarkable success of mRNA-based COVID-19 vaccines catalyzed the clinical applications of mRNA therapeutics. The current applications have spanned epidemic vaccine [19, 20], cancer vaccine [28–30], protein replacement therapy [24], cytokine therapy [31, 32], cell therapy [33, 34] and gene editing [21, 35], serving as crucial complements for traditional medical therapies. Therefore, summarizing recent advancements is essential to provide a reference for researchers and highlight future prospects of mRNA technology.

Herein, we provide a comprehensive and critical review of the latest progress of mRNA-based innovative strategies, potential challenges as well as the future development trends. We throw light on mRNA structure optimization, delivery system advancement and medical applications to illustrate the landscape of mRNA-based therapeutic strategies. We further discuss the limitations and challenges of mRNA, aiming to provide a comprehensive review for relevant research regions in post-COVID-19 era.

## Optimization of mRNA Structure

**Fig. 3:**
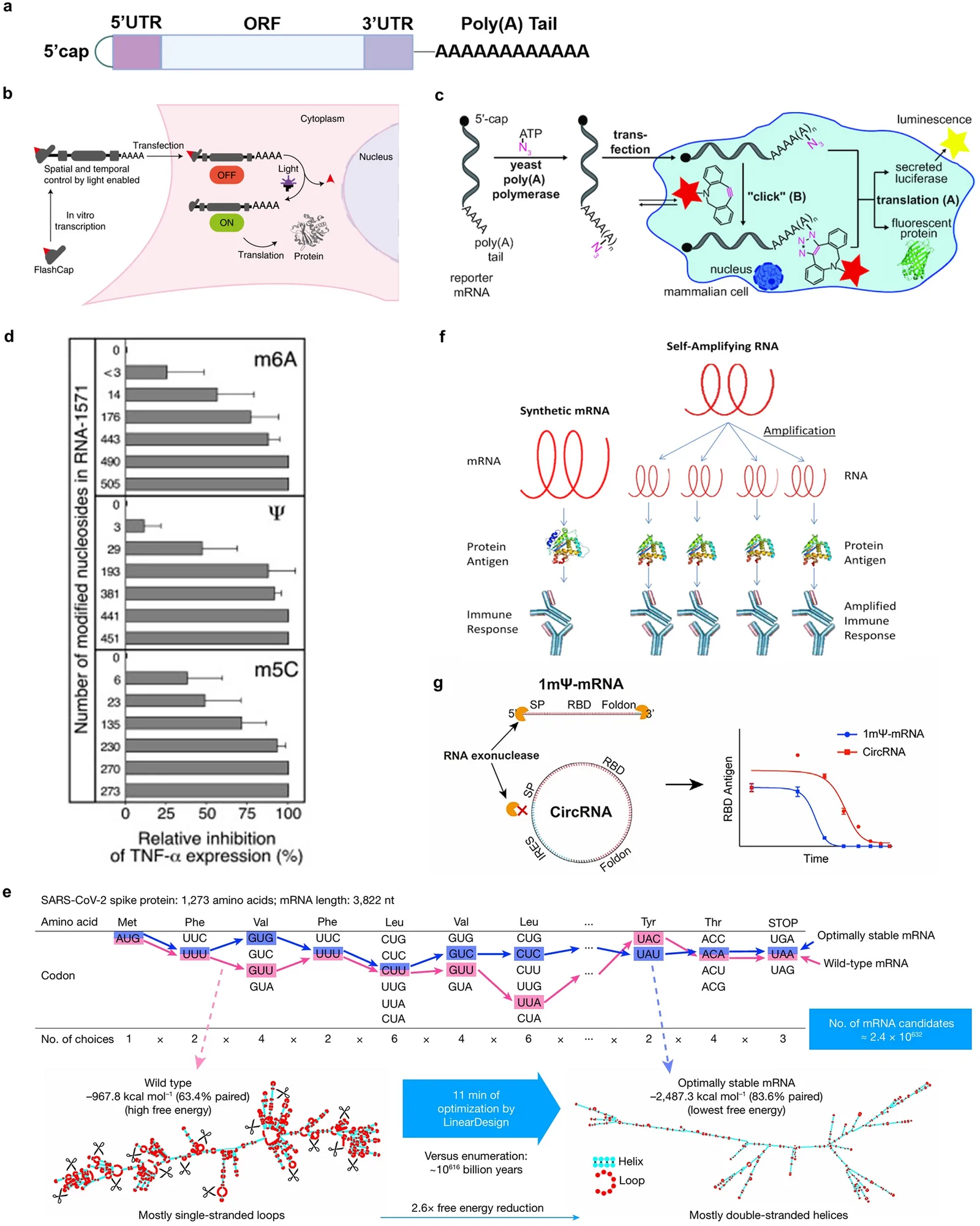
Optimization of mRNA structure. **a** General structural characteristics of artificially synthesized mRNA. **b** FlashCap modified at 5’cap was designed for optochemical control of mRNA translation. Irradiation allowed the expression of mRNA encoding eGFP with FlashCaps. Reproduced under the terms of the CC-BY license [55]. Copyright 2022, The Authors, published by Springer Nature. **c** Multiple azido-modification enabled the connection of fluorescence label with poly(A) tail through click reaction, which improved the translation efficiency and realized mRNA localization. Reproduced with permission [64]. Copyright 2019, Oxford University Press. **d** Nucleoside modification significantly reduces mRNA immunogenicity, with the reduction effect positively correlated with the number of modified nucleotides. Reproduced with permission [13]. Copyright 2005, Elsevier. **e** LinearDesign identified the optimally stable (lowest free energy) mRNA sequences in 11 min, which took 10^616^ billion years through enumeration. Reproduced under the terms of the CC-BY license [84]. Copyright 2023, The Authors, published by Springer Nature. **f** saRNA provided longer function period and enhanced translation expression with a lower dosage. Reproduced under the terms of the CC-BY license [87]. Copyright 2018, The Authors, published by Elsevier. **g** Circular structure endowed the circRNA vaccine with the resistance to RNA exonuclease and elicited higher average proportions of neutralizing antibodies in comparison with 1mΨ-modified mRNA vaccine. Reproduced under the terms of the CC-BY license [109]. Copyright 2022, The Authors, published by Elsevier.

As a nucleic acid drug, efficient protein production is essential for mRNA to intervene diseases effectively. However, the single-stranded structure of mRNA renders mRNA-based drugs inherently unstable while its intrinsic immunogenicity can easily cause in vivo clearance by immune system, which necessitates artificial optimization. Similar to naturally occurring mRNA, synthetic mRNA is also comprised of five parts: a 5’cap, a 5’untranslated region (UTR), an open reading frame (ORF), a 3’UTR and a poly(A) tail (Fig. 3a) [36]. To address above challenges, these components have been optimized to enhance mRNA stability and translation efficiency (Table 1).

**Table 1:** The impact of mRNA component optimization on mRNA immunogenicity, stability and translation efficiency

mRNA component	Strategy	mRNA immunogenicity	mRNA stability	Translational efficiency
5’cap	Enzymatic capping	Low	High	High
	m7GpppG	High	Low	Low
	ARCA	High	Middle	Middle
	CleanCap	Low	High	High
3’poly(A) tail	A long poly(A) tail	–	High	High
ORF	Codon optimization	–	–	High
UTR	Selection of UTRs from highly expressed genes/High-throughput screening	Low	High	High
Nucleoside	Nucleoside modification	Low	High	High

### 5’cap of mRNA

The mRNA cap structure in eukaryotes is an N7-methylated guanosine located at the 5’ end of the mRNA, which is linked with the first nucleotide via a reverse 5’–5’ triphosphate [37]. mRNA capping shields mRNA from exonuclease degradation, labels them for subsequent modifications (such as splicing, polyadenylation) and translocate it into the cytoplasm. The translation of mRNA cannot proceed unless the eukaryotic translation initiation factors recognize 5’cap [38]. Given the critical role of the mRNA cap, multiple capping strategies have been developed for in vitro mRNA capping. Capping enzymes, such as vaccinia capping enzyme and 2’*O*-methyltransferase [39, 40], enable in vitro capping with the addition of substrates and have been widely commercialized. The capping rate almost approaches 100% through continuous enzymatic capping optimization, while the resulting Cap1 structure represents the predominant cap form in human and murine cells, conferring low immunogenicity and high translation efficiency [41–43]. However, the process remains complicated due to the introduction of excessive enzymes. In contrast, co-transcriptional capping is a simpler way to achieve one-step capping by adding cap analogs such as m7GpppG to the reaction system [44]. However, since both ends of m7GpppG (the m7G and downstream G) can serve as initiation sites for mRNA elongation, co-transcriptional capping produces one-third to one-half of the mRNA which incorporated in the reverse orientation, leading to the failure of translation to the target protein due to lack of the entire cap structure [45, 46]. To address the problem of reverse capping, the anti-reverse cap analog (ARCA) methylates the 3’-OH group of m7G in the cap structure to ensure exclusive forward orientation incorporation into the mRNA chain. The advent of ARCA eliminates the interference from non-functional cap, yielding mRNA with improved stability. ARCA-enhanced co-transcriptional capping doubles translation in mature dendritic cells (DCs) yet remains 30% less efficient than enzymatic capping [47]. Moreover, ARCA fails to improve the low capping rate (34%–77%) caused by the competition between cap analogs and GTP during transcription initiation, prompting the development of CleanCap [48, 49]. The CleanCap achieves a capping efficiency exceeding 90%. The resulting Cap1 structure enhances mRNA stability and reduces immunogenicity, thereby improving translational efficiency [50]. Masahito et al. showed that CleanCap capping suppressed transfection-induced NF-κB activation (2.2-fold vs negative control) more effectively than ARCA (2.7-fold) [51]. Natural cap modifications, such as Cap0 and Cap1, are dependent on RNA methyltransferases, which are promiscuously active for analogs of the natural co-substrate *S*-adenosyl-l-methionine (AdoMet). Therefore, identifying co-substrate analogs of AdoMet can realize the methylation at different sites in the first nucleotide attached to the cap, which endows mRNA with various biological functions [52, 53]. Wang et.al synthesized the muti-capped mRNA, which enhanced mRNA-eIF4E-eIF4G binding and significantly increased protein expression [54]. In addition to the function of regulating mRNA stability and immunogenicity, 5’ cap modification can also take control of the initiation of mRNA translation. Nils Klöcker et al. reported 5’cap analogs with photo-cleavable groups (FlashCaps) that enabled spatial and temporal control of translation by light [55]. Different from the normal translation process, FlashCaps blocked the binding of 5’cap to eIF4E and prevented degradation of the decapping enzymes. The translation could not begin unless light-induced deprotection was acted and the origin Cap0 turned out. This innovation design facilitates light-induced translation enhancement and also maintains acceptable stability and immunogenicity compared with Cap0, which provides a novel approach for photochemical control of mRNA translation (Fig. 3b).

### 3’ poly(A) Tail of mRNA

The majority of eukaryotic mRNA 3’poly(A) tail is generated by the addition of adenines after the splicing of pre-mRNA [56]. The poly(A) tail binds to polyadenylate-binding protein (PABP), which interacts with the translation initiation factor eIF4G, and allows mRNA to form a close loop, controlling translation initiation synergistically with 5’cap [57]. The binding of PABP to mRNA can cover a length of 30 nt, which prevents mRNA from degradation by nucleic acid exonucleases, so a poly(A) tail exceeding 30 adenosines is essential for mRNA stability and translation [58]. Longer tails are generally considered to be associated with enhanced mRNA stability and expression [59–61]. While deacetylation drives poly(A) tail shortening and mRNA decay, Mroczek et al. identified that the mRNA-1273 vaccine extends mRNA half-life in macrophages through TENT5A-mediated poly(A) tail elongation [62]. Poly(A) tail elongation enhances mRNA stability, offering a novel approach to enhance mRNA vaccine performance. Additionally, mRNA with multiple chemically modified poly(A) tails was reported to maintain the translational activity of mRNA more persistently due to increased stability [63]. Additionally, the modification of the poly(A) tail enabled the tracing of the mRNA translation process. Anhäuser et al. attached multiple azido-modified adenosine nucleotides to the poly(A) tail of luciferase/eGFP mRNA through yeast poly(A) polymerase [64]. After the transfection of modified mRNA into the mammalian cell, click reaction happened with the introduction of fluorescence label (sulforhodamine B) at the 3’ poly(A) tail. The detection of fluorescence label enabled the localization of mRNA at the subcellular level. Such modification of poly(A) tail not only had no negative influence on mRNA degradation with no alteration in UTRs and coding regions, but also improved translation efficiency (Fig. 3c).

### ORF and UTRs

Beyond the 5’cap and 3’poly(A) tail, mRNA optimization primarily focuses on sequence design of the ORF and UTRs. As the coding region of mRNA, the optimization of codons in the ORF is closely related to protein yield [65, 66]. Current codon optimization is mainly based on the codon adaptation index (CAI) as the basic optimization parameter, which reflects the degree of conformity between the codons in heterologous mRNA and the optimal frequency of codon in the host cells [67]. Synonymous codons encoding the same amino acid have different tRNA abundance, and codons with higher tRNA abundance typically correspond to better translation efficiency and are used more frequently, a phenomenon known as codon usage bias. A common approach is to use synonymous codons corresponding to highly abundant tRNAs to enhance the translation rate [68].

Located flanking the ORF, UTRs harbor multiple regulatory elements that are indispensable for mRNA stability and translation efficiency, despite lacking protein-coding functions [69, 70]. On one hand, 5’UTR plays a crucial role in ribosome recruitment, scanning and translation initiation site selection. Specific 5’UTR sequences, such as the adenovirus-derived tripartite leader sequence, demonstrate superior translational efficiency compared to the 5’UTR of mRNA-1273 [71]. A-rich unstructured elements in 5’UTR have been reported to destabilize mRNA in the absence of translation [72]. On the other hand, the functions of 3’UTR are mainly determined by AU-rich elements, which are associated with mRNA inability and gene silencing mediated by miRNA [73, 74]. AU-rich elements (AREs) interact with RNA-binding proteins to promote mRNA degradation [75]. Extended AREs significantly promote mRNA degradation, whereas truncated or mutated AREs markedly enhance mRNA half-life [76]. 3’UTR harbors miRNA binding sites, facilitating the interaction between miRNA and mRNA [77]. 3’UTR lengthening increases miRNA binding sites, thereby suppressing mRNA translation. Cairns et al. demonstrated that 3’UTR extension generated 13,111 novel miRNA binding sites across 110 genes in neurons [78]. Currently, the primary means for UTR optimization includes direct selection of UTRs from highly expressed genes in host cells, such as the UTRs of human α-globin gene [79]. Another feasible method is to construct UTR libraries by deep computational modeling for high-throughput screening [80].

Moreover, the optimization of mRNA secondary structures to extend the half-life of mRNA is also a critical consideration. Highly expressed mRNA exhibits fewer secondary structures throughout the 5’UTR and the first 10 codons of the ORF [81]. Conversely, increased secondary structures formed in the remaining ORF and 3’UTR contribute to higher expression of encoded proteins. However, the formation of RNA secondary structures including double-stranded RNA (dsRNA) leads to activation of pattern recognition receptors and induces innate immune responses. One way to alter RNA secondary structure is to alter the UTR and ORF sequences. Given the functional constraints and limited flexibility in UTR design, changing the ORF sequence is a preference to regulate the secondary structure of mRNA. However, this approach tends to confound the influence of codons adjustment and mRNA structure change on protein expression. Another way is to modify the nucleosides without the change of sequences, which eliminates the effects of codon changes, such as m6A, Ψ, m1A and m5C. Nucleoside modification influences the secondary structures and interactions with proteins, which can either enhance or reduce protein expression levels [82]. More importantly, nucleoside modification affects the physicochemical properties of mRNA by inhibiting the mRNA recognition by TLRs and reducing the immunogenicity [13]. Increasing the number of modified nucleosides significantly suppresses TNF-α expression, protecting mRNA from degradation and preserving biological functions (Fig. 3d). Pseudouridine incorporation protects mRNA from exonuclease cleavage, thereby preventing the generation of short RNA fragments that activate TLR7 and TLR8 by binding to their binding pockets [83]. Meanwhile, TLR8 neglects pseudouridine as a ligand for its first binding pocket, while TLR7 neglects pseudouridine-containing RNA as a ligand for its second pocket.

### Artificial Intelligence Design for Linear mRNA

As the options for mRNA structural optimization grow exponentially with sequence extension, designing the optimal mRNA sequences through traditional enumeration and experimentation is impractical, leaving the vast majority of stable and efficient sequence designs unexplored. With the rapid advancement of AI technology, Huang et al. developed LinearDesign, an algorithm that rapidly optimizes mRNA codons and stability to identify the optimal mRNA sequence [84]. LinearDesign adapted the classical concept of lattice parsing from computational linguistics. Even in the absence of nucleoside modification, mRNA sequences generated by LinearDesign exhibited significant improvements in chemical stability and translation efficiency. The exceptional efficiency and low cost of LinearDesign position it as a critical tool for addressing future pandemics (Fig. 3e).

### Self-Amplifying RNA

Repeated infections with new coronaviruses and derived variants are a thorny issue in the aftermath of global outbreaks, while protein replacement therapy that requires multiple administrations present an additional challenge for linear non-replicating mRNA. Self-amplifying RNA (saRNA) is regarded as a promising alternative strategy.

Notably, saRNA adds the sequence of the RNA-dependent RNA polymerase (RdRp) of viral origin (such as α-viruses) and can thus regard itself as the template to generate more copies of saRNA [85]. The double-stranded structure formed during saRNA replication mimics viral RNA replication and activates pattern recognition receptors (e.g., RIG1), triggering innate immune responses and enhancing vaccine efficacy [86]. Vogel et al. first compared the saRNA vaccine with an nr-mRNA vaccine head to head and demonstrated that the former achieved comparable efficacy at lower doses with extended expression period [87]. Several influenza viruses, including H1N1 and H3N2, were tested for the effectiveness of the saRNA vaccine with satisfactory results. The authors pioneered the development of a trivalent saRNA influenza vaccine, establishing a robust foundation for saRNA researches (Fig. 3f).

Lower dose and extended prevention period are prominent advantages of saRNA, but the immune response triggered by saRNA is a “double-edged sword” since the activation of double-stranded RNA-dependent protein kinase lead to the pause of cell translation, so precise modulation of saRNA immunogenicity is essential to minimize side effects [88]. Viral non-structural proteins, namely RdRp, may unpredictably affect cellular metabolism and disrupt normal signaling pathways. Nucleoside modification has been established as an effective strategy to attenuate saRNA immunogenicity. Compared to unmodified saRNA, 5-hydroxymethylcytosine (hm^5^C)- or 5-methylcytosine (m^5^C)-modified saRNA exhibits enhanced cellular transfection efficiency by attenuating IFN response-triggered innate immunity [89]. Moreover, Remaut et al. identified co-delivery of immunosuppressive agents and saRNA purification as two additional strategies to reduce saRNA immunogenicity [90]. The authors employed cellulose-based purification to remove dsRNA byproducts, yielding high-purity saRNA preparations. The innate immune suppressor B18R, a decoy receptor for type-I IFNs, was co-delivered to inhibit interferon signaling pathways. Meanwhile, additional RdRp sequences (approximately 7000nt) increase the molecular weight of saRNA and reduce delivery efficiency, which is related to the optimization of the delivery method and carriers [91]. An alternative strategy for saRNA preparation, trans-amplification, offers a viable approach, which divides the sequence encoding the RNA polymerase and the mRNA sequence encoding the target protein into two parts and introduces them into the same cell [92].

### Circular RNA

Given the inherent instability of single-stranded mRNA, circular RNA (circRNA) emerges as a promising alternative. Generated by a non-canonical splicing event called backsplicing [93, 94], circRNA is endowed with remarkable stability due to its ring-like conformation preventing from degradation of exonuclease [95, 96]. Although natural circRNA lacks cap-dependent translation initiation structures (e.g., 5′ cap and poly(A) tail), artificial addition of a cap-independent translation initiation structure is a solution, such as internal ribosome entry sites (IRES) [97, 98]. Incorporation of a 5' cap structure enables cap-dependent translation initiation in circRNA. Abe et al. engineered a covalently linked N7-methylguanosine cap into circRNA via a branching architecture, achieving a 2–3 orders of magnitude enhancement in protein expression compared to non-capped counterparts [99]. The authors additionally achieved a 50-fold enhancement in translation efficiency through non-covalent incorporation of a cap-containing complementary short oligoribonucleotide.

Current methods for circRNA synthesis primarily include chemical ligation, enzymatic ligation or the group I intron-based permuted intron–exon method [100, 101]. In circRNA preparation, incomplete RNA cyclization may occur. Optimizing cyclization conditions can address low recovery efficiency cyclization, including linear RNA precursor concentration and ligase reaction system design. Secondary cyclization of primary ligation products enhances circularization efficiency [102]. Adding splint RNA assists cyclization by preventing intermolecular ligation and improving accuracy [103]. Du et al. developed a trans-splicing-based circRNA synthesis method [104]. The authors optimized the Mg^2+^ concentration and introduced an extended guide sequence and internal loops, significantly improving the circularization efficiency. Post-cyclization byproduct removal is critical for circRNA purification and recovery. The presence of substantial byproducts including linear RNA, triphosphorylated RNA and dsRNA triggers innate immune responses, making circRNA purification an enduring challenge. While electrophoretic separation effectively purifies research-scale products, it remains impractical for industrial manufacturing. Xiao et al. developed a scalable solution through exonuclease RNase R to selectively degrade linear RNA while preserving structurally stable circRNA [105]. Phosphatases eliminate triphosphorylated RNA residues from byproducts [104]. Additionally, Cao et al. employed microcrystalline cellulose chromatography to remove dsRNA contaminants [106]. Synergistic purification strategies enable more complete clearance of immunogenic byproducts. The combination of RNase R with high-performance liquid chromatography has been demonstrated to yield circRNA with purity exceeding 90% [97]. To improve the protein production of cirRNA, Chen et al. created a circRNA modular cloning platform to clarify the effect of each component on circRNA translation efficiency [107]. The authors applied the m6A modification in circRNA to lower the immunogenicity without the decrease of translation rate. The optimization of vector topology, 5’ and 3’UTRs, IRES and synthetic aptamers recruiting translation initiation machinery contributed to the prolonging translation period, durable function activity and exponential-increasing protein expression. Compared with linear mRNA, the integrity of IRES secondary structure is critical for circRNA functionality, while the enhancement of circRNA secondary structure can increase the stability. AI technology also takes part in the design of circRNA. Huang et al. further introduced circDesign, an algorithm platform for circRNA structure prediction and sequence design [108]. The ViennaRNA software predicted the IRES folding structure to ensure its functionality and stability. Leveraging the screened IRES sequences and LinearDesign-optimized ORFs, circRNA-designed rabies virus and varicella-zoster virus vaccines exhibited enhanced sequence stability and translational efficiency, validating the effectiveness of circDesign platform in the optimization of circRNA sequence design.

To provide protection against COVID-19 variants, Wei et al. reported a circRNA vaccine delivered by LNP [109]. Selecting the spiking protein receptor binding domain (RBD) trimers as the immunogen, the circRNA vaccine not only provided more robust and broad-spectrum protection compared to the 1mΨ-modified mRNA vaccine, but also showed better stability in mouse and rhesus monkey models, which is a valuable complement to mRNA vaccines (Fig. 3g). Beyond epidemic vaccine, circRNA has also been designed for cancer vaccines to drive immune responses in hard-to-treat malignancies such as immune exclusive tumors and immune desert tumors [110].

## Carriers for Efficient mRNA Delivery in Vivo

Owing to its high molecular weight, negative charge, and susceptibility to degradation, mRNA faces significant challenges in effectively entering target cells. As a result, naked mRNA is no longer a suitable choice, which indicates the importance of delivery vectors in mRNA transfection and subsequent protein translation [111]. To protect mRNA from degradation, delivery platforms focus on endosomal escape and targeted delivery. The scientific community has developed diverse mRNA delivery systems including platforms based on lipids, proteins/peptides, polymers and viruses, accompanied with novel delivery strategies (Table 2).

**Table 2:** Comparative analysis of mRNA delivery systems

Carrier for mRNA delivery	Advantages	Limitations	Clinical translation status
Lipid	High safety profile High transfection efficiency Composition Tunability	Immunogenicity risk	Marketed
Peptide	Favorable biocompatibility Enhanced designability Low immunogenicity of endogenous proteins	Poor stability Limited loading capacity	Early clinical trial stage
Polymer	High structural designability and controllability	Elevated toxicity	Preclinical trial stage
Viral vector	Superior transfection efficiency	Elevated toxicity Limited packaging capacity Strong immunogenicity Inherent high adjuvant property	Early clinical trial stage

### Lipid-Based Carriers

As a derivative of lipids, liposomes were first synthesized in 1965, which is a closed bilayer vesicle with a typical spherical structure [3]. When amphiphilic phospholipids are dispersed in the aqueous phase, the hydrophobic tail of the molecule tends to cluster together, avoiding the aqueous phase, while the hydrophilic head is exposed to the aqueous phase. Hydrophobic drugs are encased in a lipophilic double layer shell, while hydrophilic drugs are encapsulated in the core (water phase). Liposomes have nanoscale particle size, which are considered to be the early version of LNPs. After decades of development and the rigorous testing during the COVID-19 pandemic, LNP has emerged as the most mature and advanced carrier for mRNA delivery. The classical LNP consists of four components, including ionizable cationic lipid, cholesterol, PEGylated lipid and phospholipid, which play crucial roles in safety, mRNA stability and transfection efficiency [112].

**Fig. 4:**
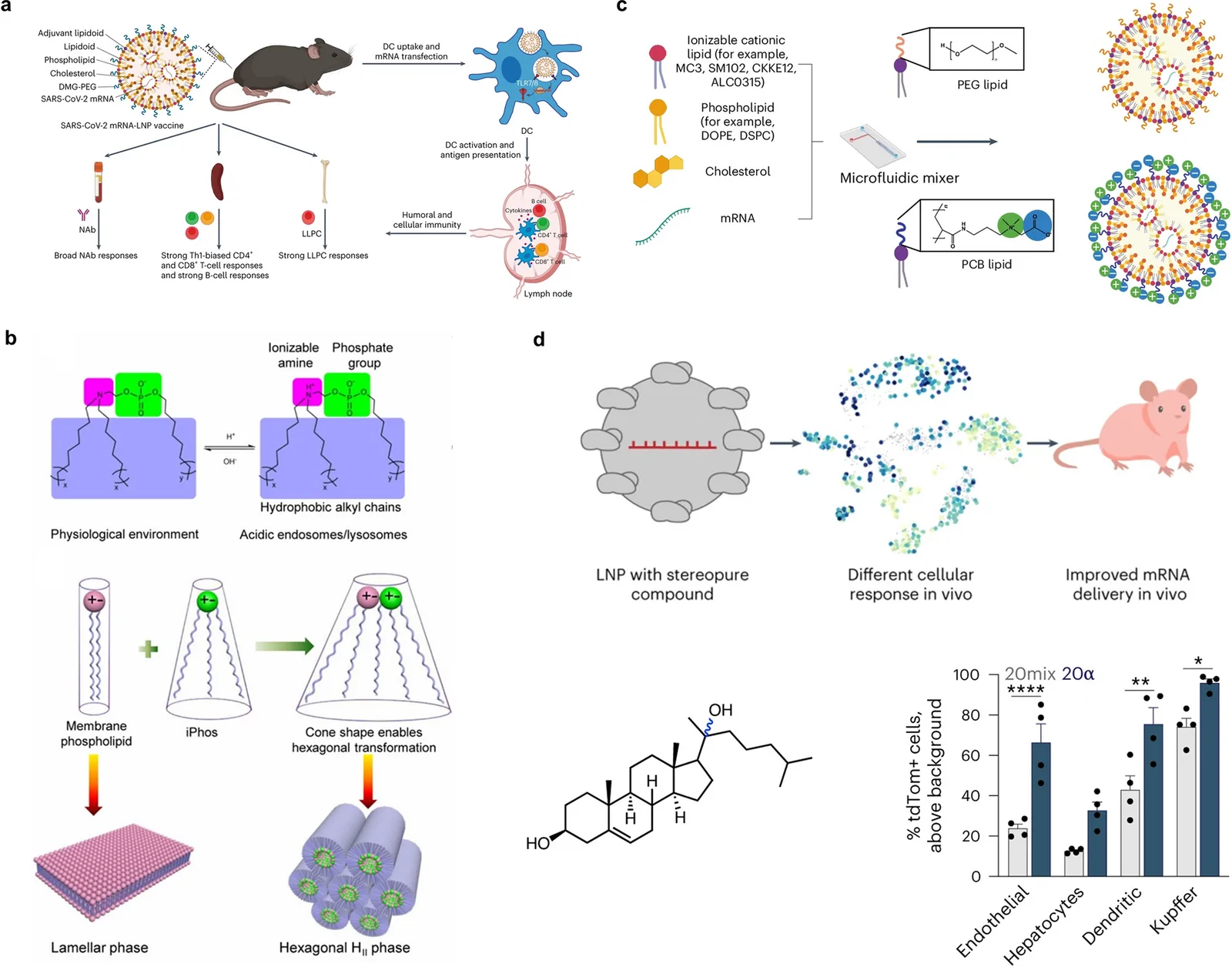
Lipid-based mRNA delivery systems. **a** TLR7/8 agonist 1 was transformed to C12-TLRa through ring-opening reaction. The LNPs were endowed with the activity of TLR7/8 agonist by replacing ionizable lipidoid with C12-TLRa. Reproduced with permission [123]. Copyright 2023, Springer Nature. **b** iPhos consisted of one ionizable amine, one phosphate group and three hydrophobic alkyl tails. In endosomal acidic environments, iPhos transitioned to a zwitterionic form, inducing hexagonal phase transitions in endosomal membranes. Reproduced with permission [128]. Copyright 2021, Springer Nature. **c** PCBs circumvented the accelerated blood clearance effect, enabling repeated dosing. Reproduced with permission [136]. Copyright 2025, Springer Nature. **d** Difference in delivery efficiency among 20-hydroxycholesterol conformers demonstrated that the spatial configuration of LNP components influences mRNA delivery efficacy. Reproduced with permission [137]. Copyright 2023, Springer Nature.

Ionizable cationic lipids contain tertiary amine groups that become positively charged at pH values below their acid dissociation constant (pKa) [113]. The acidic environment in LNP formulation ensures complete protonation of ionizable lipids, facilitating the encapsulation of anionic mRNA through charge-charge interactions. Following desorption of PEGylated lipids from the LNP surface, apolipoprotein E recognizes the exposed ionizable lipids and adsorbs onto the LNP, ultimately enabling uptake by hepatocytes with high expression of low-density lipoprotein (LDL) receptors [114]. Therefore, supplementing selective organ targeting nanoparticles with distinct surface charges can alter the adsorbed proteins, enabling tissue-specific mRNA delivery [115]. Subsequently, LNPs are encapsulated within endosomes. During endosomal maturation, the progressively acidified environment triggers extensive lipid protonation. A proposed mechanism suggests that highly cationic LNPs bind to anionic endosomal membranes, resulting in membrane destabilization and mRNA cytoplasmic release for effective transfection [116]. An alternative mechanism is the “Proton Sponge Effect” [117]. Ionizable lipids become protonated in endosomes, triggering proton pump activation. This drives massive proton influx into the endosomal compartment. To maintain charge balance, chloride ions concomitantly enter, markedly increasing osmotic pressure. The resultant water influx induces endosomal swelling and rupture, enabling mRNA cytoplasmic release. Additionally, ionizable lipids may serve as adjuvants to modulate the immunogenicity of LNPs [118]. Therefore, the screening and design of ionizable cationic lipids is crucial to LNP optimization. Currently, the primary method to select top-performed LNP is in vivo and in vitro high-throughput screening. FDA-approved ionizable cationic lipids, such as MC-3, SM-102 and ALC-0315, are all obtained through this method [119]. A high-throughput LNP screening system based on barcoded DNA has been reported, which explored combinational cation-degradable (CAD) lipid libraries and identified an optimal LNP, LNP-CAD9, for lung-targeted mRNA delivery [120]. Unsaturation, mantissa, biodegradation bond and branch chain structure are worthy of consideration in ionizable cationic lipid design [121]. A degradable skeleton represents a highly advantageous structural features in ionizable lipids, and degradable branched lipids containing long chain alkyl branches have been shown to increase mRNA delivery efficiency by three orders of magnitude [122]. To confer additional physiological functions, Han et al. partially substituted ionizable lipid with adjuvant lipidoid and endowed LNPs with TLR 7/8 activity [123]. Through a ring-opening reaction, the TLR7/8 agonist 1 was converted into C12-TLRa, preserving the TLR-stimulating capacity and introducing an ionizable amine for mRNA combination. The ionizable lipidoid in the top-performed LNP C12-113 was substituted with an increased content of C12-TLRa, while substitution with 5 mol% of C12-TLRa was identified as the optimal formulation without the change of physicochemical properties. The synthesis of C12-113/TLRa LNP exemplifies a strategy to enhance the innate immunity activation of vaccines. Compared to C12–113 LNPs, C12–113/TLRa LNPs significantly enhanced the proportion of mature DCs and elicited a more robust cytokine response (including TNF-α and IL-12p70), which consequently induced a stronger Th1-biased T cell response, neutralizing antibody production, and long-lived plasma cell responses (Fig. 4a). Rational ionizable lipid engineering has achieved targeted mRNA delivery. Xue et al. designed the siloxane-based ionizable lipid and developed the siloxane-incorporated LNP, achieving organ-selective mRNA delivery [124]. The adjustment of siloxane-based amine head and alkyl chain structures induces fine-tuning of LNP structure, enabling mRNA selective mRNA delivery to the liver, lungs, or spleen.

Cholesterol and phospholipids are two auxiliary lipids in LNP. Intercalated within the lipid bilayer, cholesterol modulates membrane fluidity and permeability, preventing mRNA leakage from the LNP core. Patel et al. developed a polymorphic eLNP with the introduction of polyhedral C-24 alkyl phytosterols through screening of natural cholesterol analogues, demonstrating that the optimization of cholesterol improved cellular uptake and endosomal escape ability of LNP [125]. Meanwhile, phospholipids play a crucial role in stabilizing LNP structure and facilitating mRNA encapsulation [126]. Modulation of phospholipid headgroups enhances interactions between LNPs and cells, promoting cellular uptake [127]. Furthermore, phospholipid design can dictate LNP organ targeting ability. Liu et al. developed ionizable phospholipids (iPhos) consisting of one tertiary amine, one phosphate group and three hydrophobic tails through the combinatorial reaction between alkylated dioxaphospholane oxide molecules and amines [128]. The hydrocarbon tail length of iPhos governed delivery tropism. Extending the carbon chain length from 9–12 to 13–16 shifted mRNA delivery from liver to spleen targeting. iPhos maintained a negative charge at physiological pH, preventing membrane fusion. After entering acidic endosomes, the tertiary amine group of iPhos undergoes protonation to form a zwitterion, which combines with three hydrophobic tails to create a conical structure. This structure induced the transition of endosomal membranes from a lamellar to a hexagonal phase preferentially, potentiating endosomal escape efficiency (Fig. 4b). This demonstrates that tunability of LNP components currently represents a pivotal enabling strategy for organ-selective mRNA delivery.

Although PEGylated lipids constitute the smallest proportion of lipid components in LNP, they significantly influence key properties of LNP such as size, dispersity and stability. During LNP formation, the PEG chains form a hydrophilic steric barrier on the particle surface that prevents aggregation, promotes uniform self-assembly, and enables precise control over LNP size and stability [129]. PEGylation confers stealth properties to LNPs, which is a well-established strategy to minimize opsonization and prolong systemic circulation [130]. Both fluorinated PEG modification and replacement of cleavable PEGylated lipids promoted cellular internalization and endosomal escape of LNP [131, 132]. However, PEGylated lipids can trigger several adverse reactions, including the induction of PEG antibodies and complement activation-related pseudoallergy [133, 134]. The presence of PEG antibodies leads to rapid clearance of the PEGylated-lipid-containing drugs upon repeated administration, namely accelerated blood clearance, which compromises therapeutic effectiveness. PEGylated lipids featuring short acyl chains exhibit accelerated dissociation from LNPs, thereby mitigating anti-PEG immune responses [135]. Additionally, identifying PEGylated lipid substitutions, such as poly(glycerol), poly(oxazolines) and polysarcosine–lipids, is a feasible way to improve the effectiveness and safety of LNP delivery. Poly(carboxybetaine)-conjugated lipid (PCB-lipid) has been validated as a functionally superior alternative to PEGylated lipid [136]. PCB, a zwitterionic polymer derived from natural glycine betaine, exhibits superhydrophilicity, minimal immunogenicity and exceptional biocompatibility, which effectively addresses the accelerated blood clearance associated with conventional PEGylated systems. Following 24h co-incubation with human PBMCs, PCB-LNPs demonstrated significantly reduced secretion of proinflammatory cytokines (IL-6, TNF-α) compared to PEG-modified counterparts. PCB-LNPs maintained consistent luciferase expression profiles across four intravenous administrations. Besides, PCB-LNPs demonstrated superior endosomal escape efficiency compared to PEG-LNPs, which may be attributed to their hydrophilicity and structural characteristics (Fig. 4c).

The optimization of components is a considerable perspective while chemical conformation has emerged as another significant factor. Hatit et al. reported that the efficacy of LNP was closely associated with stereochemistry [137]. cKK-E12 was selected for hypothesis validation, which is a lipopeptide with six stereocentres in LNP. Different batches of cKK-E12, synthesized and purified by the same method, showed varying efficiencies in mRNA delivery. Since the complicated spatial conformation of cKK-E12 hindered further exploration, 20-hydroxycholesterol, with only two conformations (20α and 20β), was selected as an alternative. The group 20mix, was prepared by combining 20α and 20β at the 2:1 molar ratio. When interference of factors including polydispersity, pKa and morphology were excluded, LNP containing 20α displayed a higher delivery efficiency. The 20mix LNPs might be more entrapped in late endosomes compared to the 20α LNPs (Fig. 4d). Although the applicability of this stereopure design to other carrier types requires further validation, this work offers a novel perspective for optimizing mRNA delivery vectors.

Surface modifications of LNPs can significantly enhance their active targeting efficiency. Antibodies, with their high specificity, are particularly well-suited for targeting strategies. Drew Weissman et al. conjugated LNPs with antibodies targeting to PECAM-1, a vascular cell adhesion molecule, achieving selective mRNA delivery to the lungs [138]. LNPs modified with CD3, CD4 and CD5 antibodies have been developed for targeted delivery to T cells [139–141]. Additionally, short peptides [142], hyaluronic acid [143] and mannose [144] are also considerable targeting alternatives. Lei et al. incorporating a mannosylated ionizable lipid in LNP for DC-targeted delivery [145]. DNA modification can also augment the targeting capabilities of LNPs. Andrew et al. demonstrated that LNPs modified with guanine-rich sequences selectively delivered mRNA to the spleen, which enhanced the uptake by class A scavenger receptors through the formation of a G-quadruplex secondary structure [146]. Aptamers, often referred to as chemical antibodies, are selected through Systematic Evolution of Ligands by Exponential Enrichment and exhibit high specificity for target ligands [147]. Lee et al. specifically deliver mRNA to tumor cells by conjugating PDL1-targeting aptamers with LNPs [148].

### Protein/Peptide-Based Carriers

**Fig. 5:**
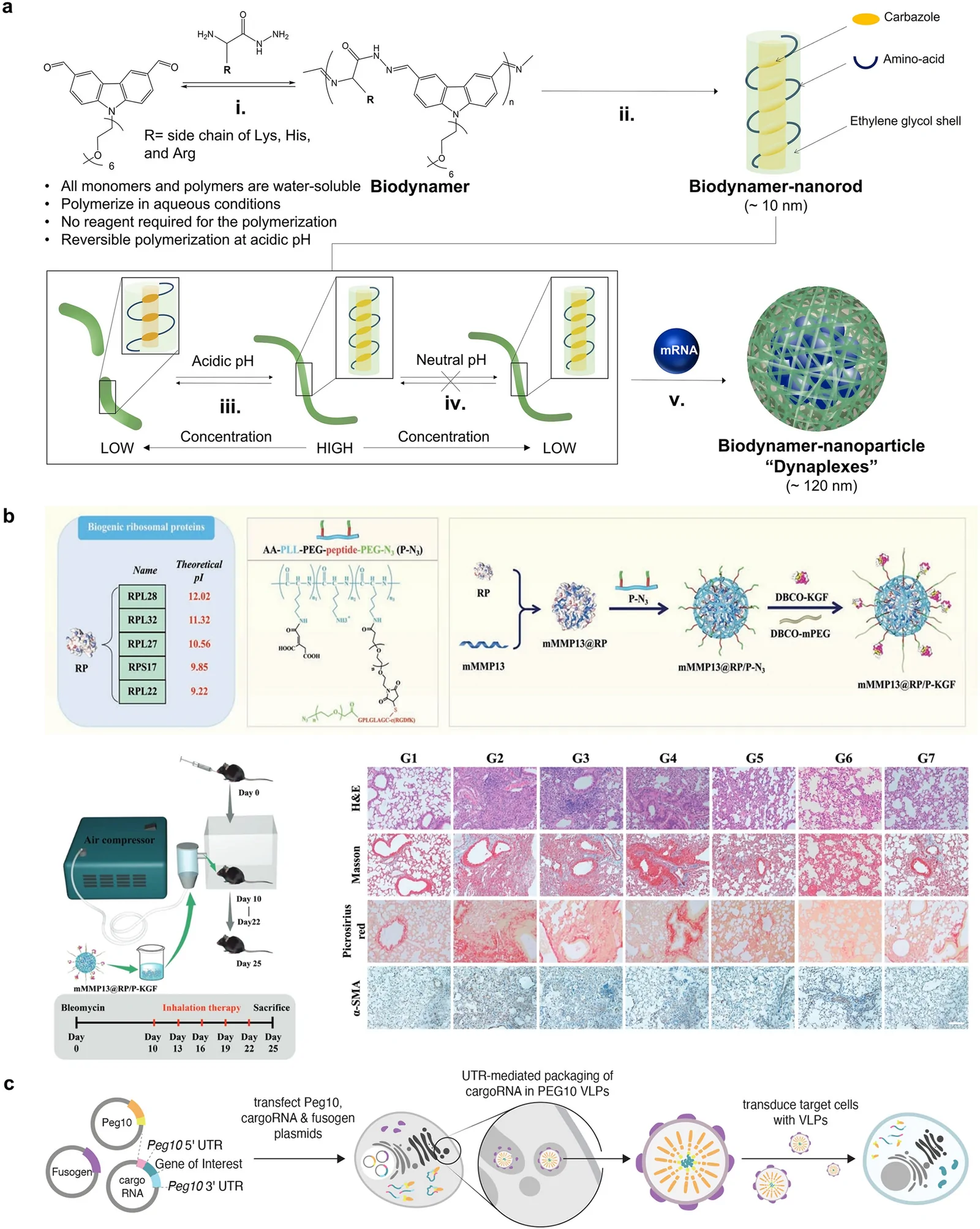
Design of protein/peptide nanoparticles for mRNA delivery. **a** pH-responsive nanorods with high transfection efficiency could realize degradation in acidic pH condition. Reproduced with permission [154]. Copyright 2023, Elsevier. **b** RP-derived mRNA vectors were constructed through click reaction and electrostatic interaction. Histological analyses of lung tissues showed the antifibrotic performance of mMMP13@RP/P-KGF. Reproduced with permission [156]. Copyright 2022, John Wiley and Sons. **c** SEND system consisted of the plasmids of Peg10, fusogen and cargo mRNA flanked with the UTRs of Peg10. The Peg10 VLP containing cargo RNA was transferred intercellularly and achieved protein expression. Reproduced with permission [157]. Copyright 2021, The American Association for the Advancement of Science.

Peptides are celebrated for their biocompatibility, designability and safety [149]. In protein/peptide-based mRNA delivery systems, self-assembly is driven by the electrostatic interaction between oppositely charged peptides and mRNA. Cell-penetrating peptides are a typical class of protein carriers for mRNA delivery. Protamine, a mixture of natural cationic cell-penetrating peptides, is an early material for mRNA delivery [15, 150]. The non-invasive cell entry mechanism of protamine is a key advantage, which avoids damage to the cell membrane [151]. Researches have produced protamine nanoparticles with different sizes by adjusting the quantity ratio of RNA and protamine [152]. Additionally, arginine-rich cell-penetrating peptides with strong positive charge also enables the delivery of mRNA [153]. Proteoid biodynamers, featuring smart strategies based on amino acid derivatives, have been developed to improve safety and transfection rate through pH-responsive nanorods [154]. Biodynamers were composed of amino acid derivatives and hexaethylene glycol-conjugated carbazole dialdehydes, polymerized in aqueous solution via dynamic covalent bonds, imines, and acylhydrazones. Positively-charged amino acid derivatives combined with mRNA via electrostatic interaction. Hexaethylene glycol-conjugated carbazole dialdehydes served as pH-responsive linkers. Under endosomal acidic conditions, protonation of the nitrogen atom on the carbazole ring promoted endosomal escape and dissociation of biodynamers, releasing mRNA into the cytoplasm. Meanwhile, degradation of biodynamers reduced the cellular accumulation, thereby mitigating potential biotoxicity. Dynaplexes were endowed with a 3-time transfection rate than conventional transfection agents, alongside exceptional biocompatibility as a non-viral mRNA delivery platform (Fig. 5a). However, the efficacy of Dynaplexes has only been validated in cellular and zebrafish models, warranting further investigation further evaluation of their efficiency and safety in murine or large animal models.

The intrinsic adjuvant property of protamine, along with the excessive binding to mRNA, has a detrimental effect on mRNA expression [155], while endogenous proteins can reduce the occurrence of heterogeneous cross-reactions. Zhang et al. utilized ribosomal protein (RP) to design an inhalation nanoparticle that co-delivered the mRNA of matrix metalloproteinase 13 (mMMP13) and keratinocyte growth factor (KGF) into fibrotic lung tissue to reverse pulmonary fibrosis [156]. The delivery vector was composed of RP-condensed mMMP13 cores, a bifunctional peptide-modified corona (AA-PLL-PEG-c (RGDfK)), and KGF with a PEGylated shielding shell, assembled via electrostatic interaction and click reaction. The bifunctional peptide-modified corona targeted myofibroblasts and injured alveolar epithelial cells with high integrin expression via the RGD motif, while also possessing pH-triggered charge reversal capability to facilitate endosomal escape. KGF was responsively released in fibrotic lesions with high MMP2 concentrations, avoiding nonspecific effects on normal tissues. The combined delivery of mMMP13 and KGF achieved fibrotic alveolar reconstruction, reversing pulmonary fibrosis (Fig. 5b). While RP is an intracellular protein, its extracellular delivery may trigger innate immune responses. Additionally, the multiple protective layers may hinder efficient mRNA release.

In addition to ribosomal proteins, numerous endogenous proteins encoded in the human genome are viable candidates for mRNA delivery. The Selective Endogenous eNcapsidation for cellular Delivery (SEND) system is a successful peptide-based modular platform to deliver specific mRNA, comprising Peg10, cargo RNA and a fusogen (virus envelope protein) [157]. Peg10, a long terminal repeat retrotransposon homolog protein, binds UTR sequences of its own mRNA to form virus-like particles (VLPs) and delivers them intercellularly via extracellular vesicles. Therefore, the authors inserted the UTR sequence of Peg10 mRNA into the cargo mRNA, enabling the plasmid-translated Peg10 to bind the cargo RNA and assemble into VLPs. The fusogenic protein facilitated VLPs to realize cell entry. As Peg10 is an endogenous protein in mammals, the SEND system exhibits low immunogenicity, which is a potential tool for gene treatment (Fig. 5c). The low endogenous expression of Peg10 necessitates exogenous overexpression via plasmids for efficient mRNA delivery, which may lead to difficulties in clinical translation. Human paraneoplastic antigen Ma2, another endogenous protein, was also been identified as a feasible vector for mRNA delivery with the formation of icosahedral capsids [158].

###  Polymer-Based Carriers

Due to structural designability and controllability, plentiful of polymer-based nanoparticles have been developed for disease intervention in recent years, driving significant advancements in polymer-based mRNA delivery platforms. Although less safe and efficient than LNP, polymers offer unique advantages in carrier design for special functions.

**Fig. 6:**
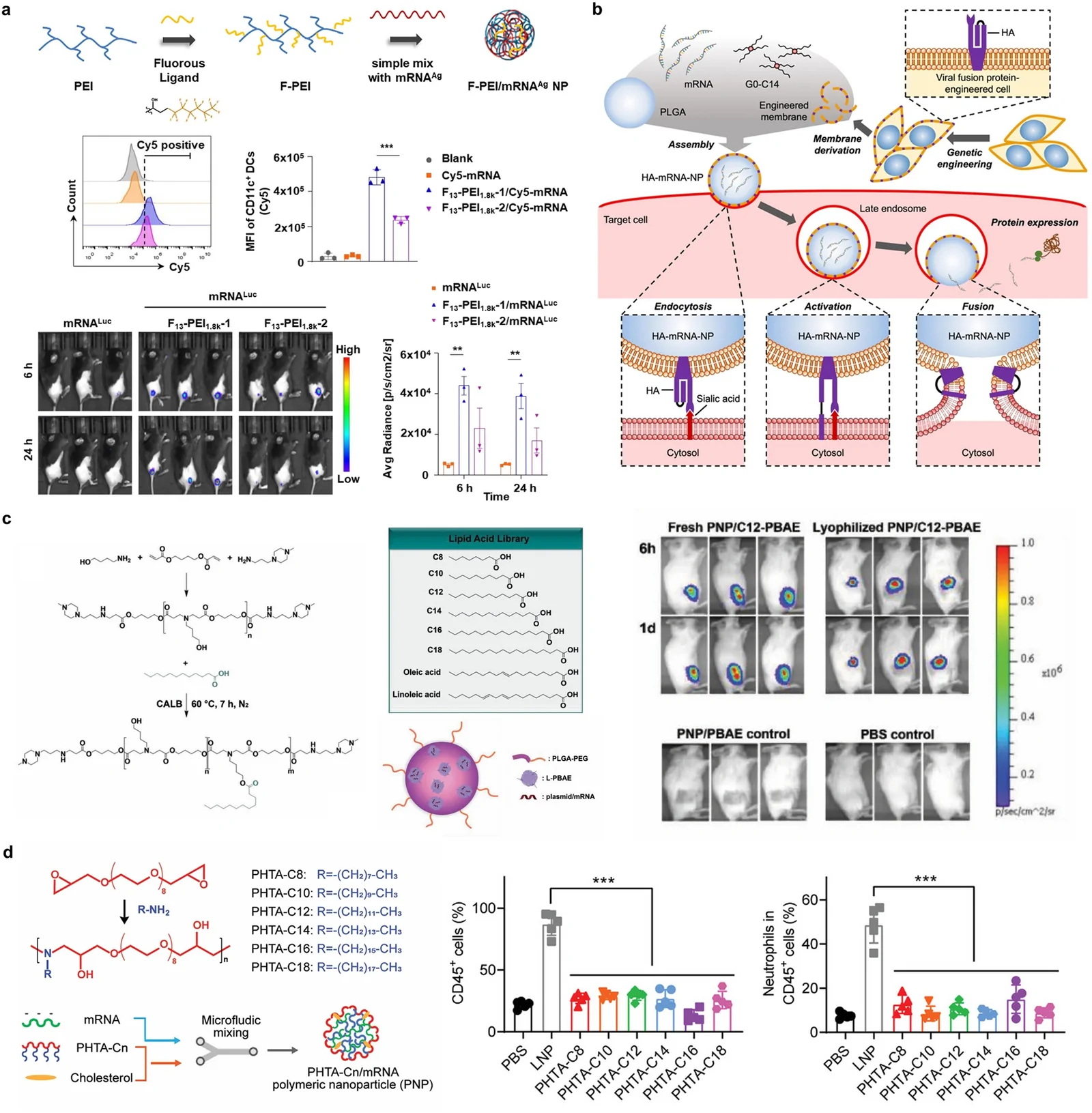
Illustration and preparation of mainstream polymer carriers. **a** Personalized cancer vaccine F-PEI/mRNA^Ag^ was synthesized by grafting fluoroalkanes to PEI, which showed high mRNA delivery efficiency both in vitro and in vivo. Reproduced with permission [164]. Copyright 2023, Elsevier. **b** Cell membrane expressed HA was coated on PLGA to achieve virus-mimicking endosomal escape. Reproduced with permission [166]. Copyright 2022, John Wiley and Sons. **c** “Particle-in-Particle” structure was achieved through the self-assembly between lipid-modified PBAE and PLGA-PEG. The lyophilized particles showed comparable transfection efficacy with the fresh particles after storage at − 20 °C for 12 months. Reproduced with permission [169]. Copyright 2022, John Wiley and Sons. **d** One-pot amino-epoxy ring-opening polymerization method was applied to the formation of PHTA with HTA repeating units. In mRNA delivery, PHTA induced minimal systemic inflammation. Reproduced with permission [171]. Copyright 2023, John Wiley and Sons.

PEI, an organic branched or linear polyamine polymer [159], is one of the common polymer carriers in mRNA delivery. The large amount of positive charge endows PEI with a strong endosomal escape ability, but leads to poor biocompatibility inevitably. Many PEI-related optimization focus on molecular weight, branching, buffer, oligonucleotide structure and method of preparation [160]. Other effective modifications including deoxycholic acid-conjugated PEI for enhanced hydrophilia [161], hydrogel consisting of graphene oxide and PEI for increased drug-loading efficiency [162], and cyclodextrin-PEI conjugate for more drug permeation [163]. Additionally, Li et al. developed a novel polymer called F-PEI for personalized mRNA cancer vaccines, which was synthesized by grafting fluoroalkanes to PEI with low molecular weight and low cytotoxicity [164]. The fluorinated modification rendered F-PEI amphiphilic, endowing F-PEI with a strong ability to penetrate the lipid bilayer of cell membranes and endosomal membranes. The authors utilized F-PEI to deliver mRNA encoding fluorescent proteins, demonstrating high delivery efficiency both in vitro and in vivo. Simple mixing of F-PEI and mRNA encoding neoantigens yielded F-PEI/mRNA^Ag^, which activated TLR4 and promoted the activation of antigen presenting cells (APCs) without additional adjuvants, effectively suppressing B16-OVA melanoma growth (Fig. 6a). However, F-PEI/mRNA^Ag^ delivery lacks tumor-targeting specificity, leading to off-target mRNA expression in non-cancerous cells, which compromises therapeutic efficacy and increases potential side effects.

Polyesters represent another class of mRNA polymeric carriers. Incorporating labile chemical bonds, including carbonate, ester, amide, and phosphate linkages significantly enhances the biodegradability and biocompatibility of polyesters. Among these, poly (lactic-co-glycolic acid) (PLGA) is a FDA-approved polyester with excellent safety, stability and delivery efficiency [165]. The electrically neutral nature of PLGA necessitates cationic modifications for effective mRNA delivery. Zhang et al. developed a virus-mimicking cell membrane-coated nanoparticle to deliver mRNA to the cytoplasm [166]. The hemagglutinin (HA) protein on the surface of influenza A virus mediates viral envelope fusion with surrounding membranes at endosomal pH. Therefore, the authors induced high expression of HA on the membrane of B16F10 cells through plasmid transfection. These cell membranes were then stripped and coated onto PLGA nanoparticle cores, which loaded with mRNA with the help of the cationic lipid-like molecule G0-C14. This carrier utilized cell membrane coating technology and genetic engineering to achieve efficient virus-mimicking endosomal escape, yet its complex fabrication process poses challenges for industrial-scale production (Fig. 6b). Poly(β-amino ester) (PBAE) is another biodegradable mainstream polyester for mRNA delivery [167, 168]. Li et al. developed a “Particle-in-Particle” carrier for mRNA COVID-19 vaccines [169]. mRNA was encapsulated in lipid-modified PBAEs (L-PBAEs) through electrostatic interactions. The lipid modification enhanced mRNA delivery efficiency by facilitating nanoparticle-cell membrane fusion, while the increased hydrophobicity of PBAE improved carrier stability at the cost of elevated cytotoxicity. The self-assembly between L-PBAEs and PLGA-PEGs generated a “Particle-in-Particle” nanostructure that protected mRNA from degradation and enabled sustained release. The vaccine could maintain its remarkable property and function for at least 12 months of storage at −20 °C (Fig. 6c). Robert Langer et al. demonstrated that hyperbranched PBAE (hPBAE) was particularly suitable for the nebulized pulmonary delivery of mRNA [170]. The hyperbranched structure exhibited greater stability and lower dispersion compared to the linear structure. Repeat dosing of hPBAE-mRNA realized consistent protein production without local or systemic toxicity.

In addition to PEI and polyesters for mRNA delivery, the synthesis of PHTA addresses the inflammatory side effects of mRNA vaccines [171]. PHTA was synthesized via one-pot amino-epoxy ring-opening polymerization method, forming a class of alternating copolymers containing ortho-hydroxy tertiary amine (HTA) repeating units. The PEG backbone and alkyl side chain in PHTA polymers could condense mRNA, stabilize polymeric nanoparticles (PNPs) and prolong circulation period. Neither intradermal, intranasal nor intravenous delivery of PNP elicited significant leukocyte infiltration, inducing only moderate upregulation of serum cytokines sufficient for T-cell activation while circumventing LNP-associated cardiomyocyte apoptosis. The authors attributed this immunomodulation to hydroxyl groups in PHTA repeating units chelating redox-active metal ions (such as Fe^2+^), suppressing ROS generation. PNP successfully confined inflammatory responses within the narrow therapeutic window that balanced optimal T-cell activation with minimal systemic inflammation, providing a safer alternative platform for mRNA vaccination (Fig. 6d).

In the pursuit of targeted mRNA delivery, Anderson et al. co-formulated PBAE with PEG lipids to enhance its stability in serum, developing the first biodegradable polymeric nanoparticles for systemic mRNA delivery selectively to the lung [172]. Leveraging the lung-targeting property of PBAE, Kim et al. delivered mRNA encoding anti-VEGF antibodies for the treatment of lung cancer [173]. Additionally, Anderson et al. demonstrated that the modification of PBAE side chains with caprolactone enabled mRNA delivery to the spleen through intravenous administration [174]. The incorporation of ligands enhances the targeting capabilities of polymeric carriers, enabling active targeting. Dong et al. modified the carriers with cyclic Arg-Gly-Asp peptides, achieving targeted delivery to tumor cells with high integrin receptor expression and enhancing tumor site accumulation [175]. The modification of mannose and CD8 antibodies were also designed for DC and T cell targeting, respectively [176, 177]. Moreover, pH-responsive polymeric carriers have emerged as a promising strategy for tumor targeting due to the unique acidic pH of tumors. Zhang et al. developed a pH-responsive vector through the combination of acetalated cyclic oligosaccharide (ACD) and PEI [178]. ACD hydrolyzed into water-soluble molecules under acidic conditions, enabling responsive mRNA release and tissue-specific gene editing at the tumor sites.

In addition to pH-responsive delivery for targeting purposes, glutathione (GSH)-responsive systems represent another important class of stimuli-responsive mechanisms, enabling mRNA release in the cytoplasm where GSH concentrations are elevated. The disulfide bonds incorporated into the polymer undergo cleavage upon reaction with GSH, thereby triggering the release of mRNA from the carrier into the cytoplasm [179]. Furthermore, adenosine triphosphate (ATP), as an energy carrier, exhibits higher intracellular concentrations compared to the extracellular environment. Kazunori et al. developed ATP-responsive polyplex micelles by complexing mRNA with poly(ethylene glycol)-polycation block copolymers derivatized with phenylboronic acid and polyol groups [180]. The phenylboronate ester linkages spontaneously formed within micelles underwent cleavage in high-ATP environments, enabling controlled mRNA release.

### Viral Vectors

As the core tool for gene editing, viral vectors can overcome in vivo delivery barriers after natural evolution, exhibiting remarkable nucleic acid delivery capabilities. Adenovirus, a double-stranded DNA virus, is a commonly utilized viral vector for SARS-CoV-2 vaccine development [181, 182], but limited research utilized adenoviruses for mRNA delivery. In contrast, lentiviruses have been engineered for mRNA delivery. Cai et al. co-delivered Cas9 mRNA and sgRNA (single guide RNA) targeting vascular endothelial growth factor A (VEGF-A) through lentiviral vectors, achieving the prevention of wet age-related macular degeneration in mice (mLP-CRISPR system) [183]. The MS2 coat (MS2C) protein incorporated into lentivirus encapsulated mRNA specifically by binding to the MS2 stem loop inserted into the mRNA sequence. Since anti-VEGF agents, the first-line treatment for age-related macular degeneration, required repeated invasive injections, mLP-CRISPR system achieved a 44% knockout of the VEGF-A gene with one single subretinal injection. Similarly, based on the MS2C-mediated mRNA encapsulation strategy, Cai et al. also employed a lentivirus carrying Cas9 mRNA and viral-gene-targeting sgRNA to cure herpetic stromal keratitis in murine models (HELP system) [184]. The VSV-G envelope protein coating HELP enabled infection of neurons with broad cellular tropism and facilitated retrograde axonal transport mediated by cytoplasmic dynein. Consequently, herpes simplex virus type 1 (HSV-1) infection in the cornea and neurons was blocked effectively due to the inhibition of HSV-1 replication.

Since lentiviral vectors are ranked as biosafety level (BSL) 2 or 2+ classification, the adeno-associated virus (AAV) is regarded as a safer vector with its BSL-1 classification. Given that AAV requires inverted terminal repeats (ITRs) as the signal for DNA packaging, Yang et al. also introduced the MS2C protein, which binds to the MS2 stem loop inserted into the mRNA sequence, serving as the RNA-packaging signal [185]. This approach aligns with the aforementioned mRNA assembly in lentiviral. AAV was thus transformed into an RNA-packaging viral vector, which successfully crossed the blood–brain barrier (BBB) and achieved whole-brain mRNA delivery.

However, the strong immunogenicity and inherent high adjuvant property of viral vectors lead to clearance by the immune system and reduced delivery efficiency [186]. Additionally, the improvement in delivery efficiency results in dose-limiting toxicity issues, so researches on viral vectors is relatively limited in competition with mainstream delivery systems.

### Innovative Platforms for mRNA Delivery

####  Extracellular Vesicle

**Fig. 7:**
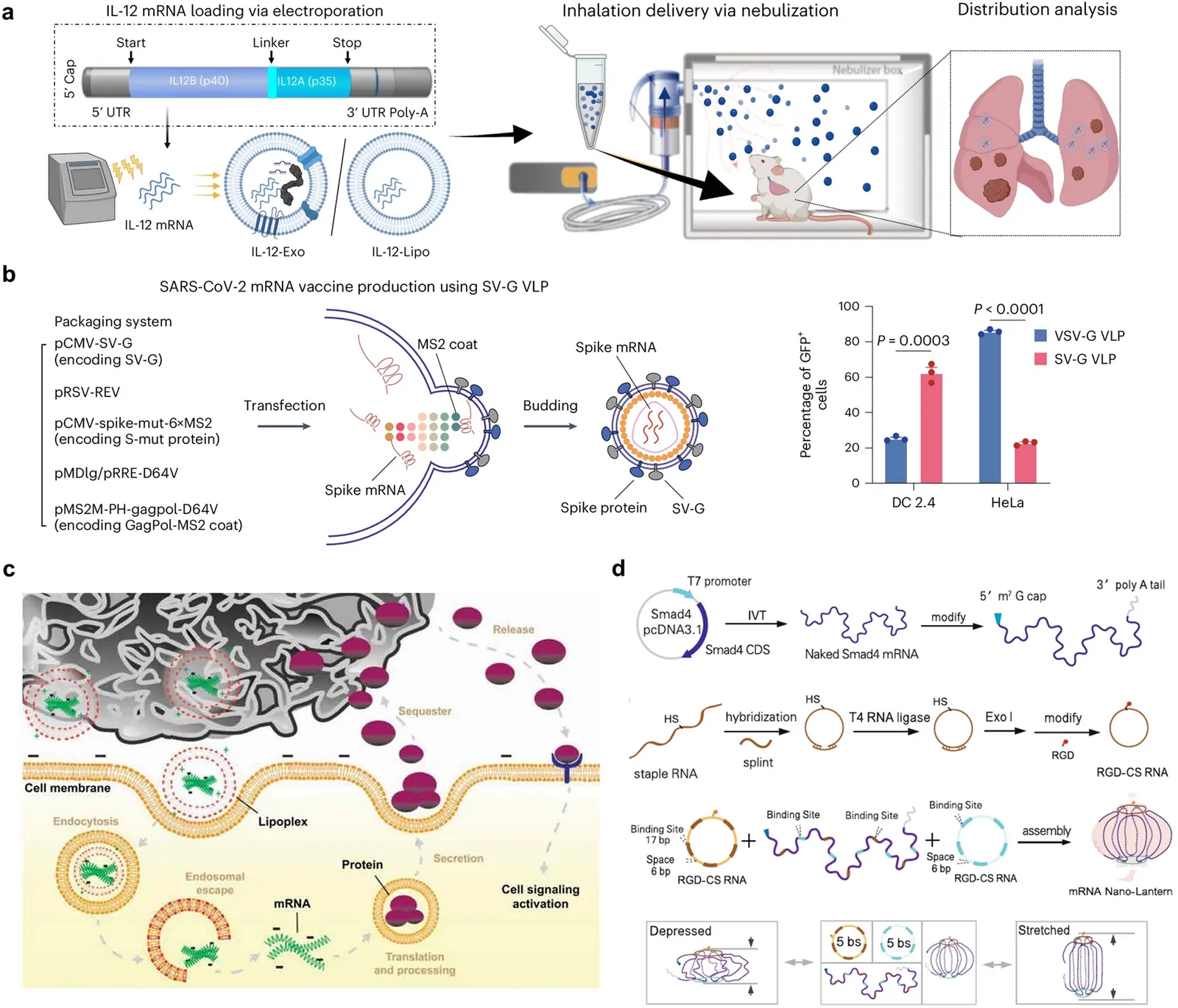
Principle of new types of vectors for mRNA delivery. **a** Electroporation realized the entrance of IL-12 mRNA into EVs for lung carcinoma therapy. The expressed IL-12 promoted the infiltration of immune cells in lung. Reproduced with permission [192]. Copyright 2023, Springer Nature. **b** Spike mRNA was encapsulated in VLPs through the interaction with coat protein during VLP self-assembly and SV-G endowed the nanoparticle with DC-targeted capability. Reproduced with permission [204]. Copyright 2024, Springer Nature. **c** MCMs sequestered the protein expressed by mRNA and prolonged the function period of protein. **d** Only two CS-RNAs were required to self-assemble the lantern-shaped RNA origami structure, achieving efficient mRNA delivery. Reproduced under the terms of the CC-BY license [219]. Copyright 2023, The Authors, published by Springer Nature.

While traditional mRNA delivery carriers are flourishing, novel delivery methods are springing up simultaneously. For example, extracellular vesicle (EV) has been studied as a promising mRNA delivery platform in recent years. EVs are nanoscale vesicles secreted by cells, mediating intercellular communication and facilitate the exchange of proteins, lipids and genetic material [187–189]. Cell-derived EVs exhibit remarkable biocompatibility and low immunogenicity, while functional and activity variations of EVs between different batches may influence the delivery efficacy. EVs from different cells varies in properties and functions. For instance, EVs from human embryonic kidney (HEK293) cells are characterized by low immunogenicity and high transfection efficiency [190]. Due to the small size of EVs, loading siRNA and microRNA through EVs is a more common approach, while loading long mRNA into EVs remains challenging. Cheng et al. developed an inhalable dry powder mRNA vaccine based on lung-derived EVs [191]. Red fluorescent protein (RFP) mRNA was transferred into EVs and liposomes through electroporation, generating RFP–Exos and RFP-Lipos, respectively. Compared to RFP-Lipos, RFP-Exos exhibited superior cellular uptake and parenchymal distribution in bronchioles and parenchyma, which penetrated lung mucus efficiently. The authors utilized EVs to deliver mRNA encoding SARS-CoV-2 spike protein, which induced significantly stronger IgG and secretory IgA antibody responses. Furthermore, lyophilized EVs remained stable at room temperature and elicited effective antibody protection for 28 days. Two years later, Cheng et al. extended the EV-based mRNA delivery system to the treatment of lung cancer [192]. Interleukin-12 (IL-12) mRNA was loaded into human embryonic kidney cell-derived EVs by electroporation and delivered through inhalation. Compared with IL-12-Lipos, IL-12-Exos exhibited superior lung accumulation and reduced off-target side effects. IL-12-Exos delivered IL-12 mRNA to the lung tumor microenvironment, inducing localized IFNγ production while avoiding systemic inflammation associated with elevated serum IFNγ levels. IL-12-Exos enhanced infiltration of CD8 + T cells, NK cells, and CD4 + T cells in the pulmonary tumor microenvironment, potentially converting immunologically cold tumors into hot tumors. However, the high production cost of IL-12-Exos limits their scalability for mass manufacturing, while their excellent biocompatibility and non-invasive administration route demonstrate notable translational potential (Fig. 7a). Additionally, EVs possess unique capabilities to penetrate biological barriers, such as crossing the blood–brain barrier (BBB), offering new avenues for the treatment of central nervous system diseases. Jiang et al. developed engineered leukocyte-derived EVs to deliver mRNA into neurons [193]. During neuroinflammation, upregulated leukocyte adhesion molecules on brain microvascular endothelial cells enhance BBB permeability, facilitating the entry of circulating leukocyte-derived EVs into the brain. Consistent with Peg10, the activity-regulated cytoskeleton-associated protein (Arc) exhibits evolutionary homology to retroviral capsid proteins. The addition of Arc protein capsids to EVs and the insertion of Arc 5’UTR to the cargo mRNA improved the stability of EVs and increased mRNA loading capacity.

#### Polyphenol

In addition to EVs, natural compounds such as polyphenols have also been explored as mRNA carriers [194]. Polyphenols, abundant in plants, not only show remarkable effects in the treatment of bacteria [195], tumor [196, 197], virus [198] and inflammatory [199–201], but also demonstrating potential for mRNA delivery due to their excellent biocompatibility. Chen et al. reported an IL-10 mRNA delivery system based on polyphenol for the treatment of ulcerative colitis [202]. Polyphenol ellagic acid combined with mRNA through supramolecular binding, providing nuclease protection. The addition of PEI conferred a positive charge to the system, enabling subsequent modification with hyaluronic acid and bilirubin. Hyaluronic acid selectively targeted CD44-overexpressing colon epithelial cells and colonic macrophages, while bilirubin protected hyaluronic acid from enzymatic degradation. Although multiple modification protected mRNA effectively, challenges in mRNA release led to lower protein expression efficiency than mainstream carriers.

####  Virus-like Particle

Virus-like particles (VLPs) are intermediates between viral and non-viral vectors that contain viral vector components, including the envelope and capsid, but lack the viral genome, which eliminates the risk of infection and genome integration [186]. Under specific conditions, viral structural proteins can self-assemble into VLPs. While VLPs lack targeting capabilities inherently, they can be engineered for targeted delivery through modifications of surface proteins, namely “pseudotyping” [203]. The VLP with DC targeting capability have been developed [204]. The MS2 stem-ring structure inserted between the stop codon and the poly(A) tail in mRNA could interact with the MS2C protein fused to the lentivirus GagPol polyproteins, which enabled mRNA to be internalized during the self-assembly of lentivirus GagPol polyproteins into virus-like vectors. The authors engineered Sindbis virus glycoprotein (SV-G) to replace vesicular stomatitis virus G protein with broad-spectrum affinity, which endowed VLPs with DC targeting capability by recognizing DC-specific intercellular adhesion molecule 3-grabbing non-integrin. Preparatory work for antitumor clinical trials based on this vaccine technology has commenced, positioning it as a promising next-generation platform for viral infection and tumor treatment (Fig. 7b). However, VLP production platforms primarily base on bacteria, yeast and mammalian cells, so the variability between batches poses a challenge for standardized manufacturing [205]. Additionally, VLPs retain the three-dimensional structures and surface antigen epitopes similar with those of natural viruses, enabling immune recognition as pathogens and triggering robust immune responses, which compromises mRNA delivery [206].

#### Inorganic Nanoparticle

Despite the lower maturity and narrower application breadth compared with LNP and polymers, inorganic nanoparticles are also promising for mRNA delivery since carbon quantum dots (CQD) [207], gold nanoparticles [208] and mesoporous silica nanoparticles [209] are all typical successful examples. CQDs possess small sizes but large specific surface areas, which realizes rapid cellular uptake. However, CQDs with large diameters cannot be cleared by the kidneys [210]. The unique photoluminescent property of CQDs enables in vivo observation and the tracking of mRNA delivery [211]. Besides, gold nanoparticles can realize the regulation of biosafety and targeting properties through adjustments in size and surface modifications. The unique optical characteristic allows them for photothermal and photodynamic therapies simultaneously during mRNA delivery [212]. Additionally, the classic spherical structure of mesoporous silica nanoparticles endows them with exceptional loading capacity, while their stable mechanical, thermal and chemical properties prevent the premature release of mRNA during transport [213]. Moreover, Khalil et al. developed mineral-coated microparticles (MCMs) to deliver mRNA encoding basic fibroblast growth factor for the improvement of wound healing in a murine diabetic wound model [214]. Hydroxyapatite powder was incubated in modified simulated body fluid for 5 days and MCMs were formed with calcium phosphate coatings. The plate-like coating nanostructure endowed MCMs with the ability of sequestering and stabilizing large amounts of rapidly generated proteins, so the biological response was prolonged due to the sustained protein release (Fig. 7c). MCMs were also applied to promote hindlimb function in the treatment of spinal cord injury [215]. The high expression of therapeutic protein Chondroitinase ABC decreased the sedimentation of chondroitin sulfate proteoglycan, which impeded spinal cord injury recovery.

#### RNA Origami

DNA origami is widely recognized for its ability to deliver drugs and nucleic acids [216–218], while RNA can also be engineered to deliver mRNA. Hu et al. utilized RNA origami to fold RNA into a flexible lantern shape, so target mRNA could be delivered to prohibit the deterioration of colorectal cancer [219]. Circular staple RNAs (CS-RNAs) were synthesized through the self-link of liner staple RNA via T4 RNA Ligase, modified with RGD (Arg-Gly-Asp) peptide to target integrin αVβ3-overexpressing colorectal cancer cells. Two CS-RNAs anchored the mRNA by complementary base pairing at binding sites, extending its half-life by conferring nuclease resistance. The rigid double-stranded architecture of conventional RNA origami impedes mRNA dissociation and translation, whereas the lantern-shaped flexible RNA origami maintains predominantly single-stranded mRNA regions, enabling direct ribosomal access and efficient protein synthesis. While the RNA lantern design shows conceptual promise, critical challenges remain in optimizing mRNA stability and relaxing the stringent binding site requirements (Fig. 7d).

## Applications of mRNA-Based Therapeutics

The core of mRNA therapeutics lies in the delivery of mRNA encoding specific proteins via vectors, the translation into proteins in ribosomes following endosomal escape, and the subsequent functional execution. mRNA played a pivotal role in the global fight against COVID-19, highlighting the remarkable advantages of mRNA-based therapeutics. In terms of safety, mRNA vaccines eliminate the risk of exogenous gene insertion, as the translation process occurs in the cytoplasm. In the development of vaccines, mRNA vaccines only require delivery of the pathogen’s characteristic genetic sequence, reducing infection-related side effects compared with inactivated and live attenuated vaccines that deliver inactivated or attenuated pathogens. Coupled with the flexible design and efficient production, mRNA therapeutics have been widely applied in the fields of epidemic vaccine, cancer vaccine, protein replacement therapy, cytokine therapy, cell therapy and gene editing.

### Epidemic Vaccine

**Fig. 8:**
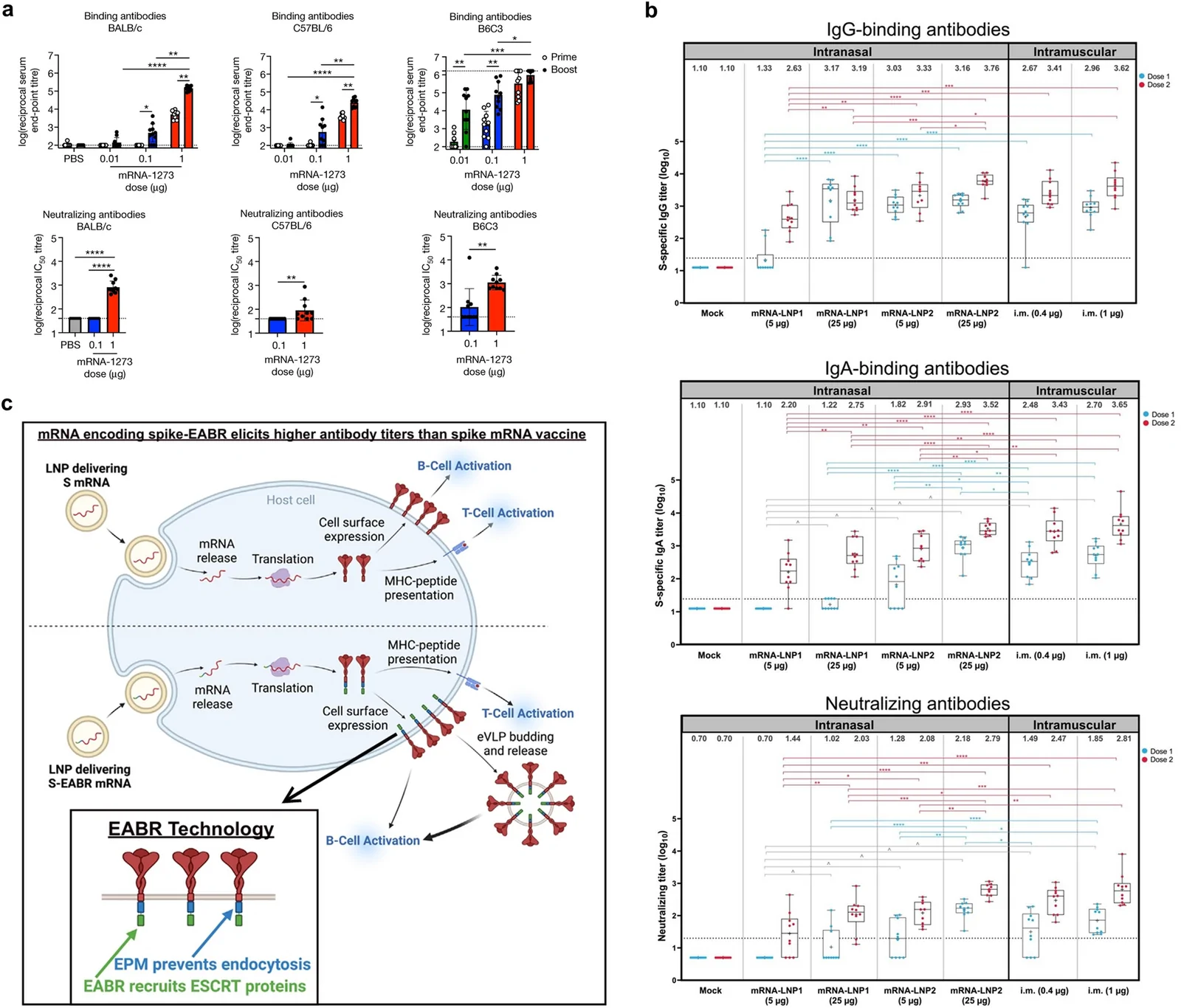
Typical epidemic vaccines and derived optimization strategy. **a** Robust immunoreaction of mRNA-1273 was activated in various murine strains. Reproduced with permission [19]. Copyright 2020, Springer Nature. **b** Intranasal mRNA vaccine elicited high S-specific serum antibody titers comparable to intramuscular controls in hamsters. Reproduced under the terms of the CC-BY license [223]. Copyright 2023, The Authors, published by the American Association for the Advancement of Science. **c** EABR technology endowed mRNA vaccine with the capability of endocytosis prevention and ESCRT protein recruitment, which activated robust immune response in vivo. Reproduced with permission [236]. Copyright 2023, Elsevier.

The approval of mRNA vaccines against COVID-19 during COVID-19 pandemic marked a quantum leap, which not only alleviated the enormous pressure of virus transmission but also accelerated the clinical application of mRNA epidemic vaccines. In 2020, BNT162b2 received emergency authorization from the FDA, becoming the first mRNA-based drug approved for human use [20]. In the phase III clinical trial, BNT162b2 demonstrated an overall preventive efficacy of 95%. Consistent with BNT162b2, mRNA-1273, developed by Moderna to encode a SARS-CoV-2 spike protein that stabilizes in a prefusion conformation, was highly successful in COVID-19, contributing to rapid advances in mRNA technology [19]. Delivered by LNP, mRNA-1273 was injected intramuscularly. The serum of mice was collected after 2 weeks and neutralizing antibodies induced by SARS-CoV-2 pseudovirus were detected, showing a dose-dependent response. Additionally, the immune reaction elicited by mRNA-1273 was assessed to be a balanced Th1/Th2 response rather than the Th2-biased response, which avoided the vaccine-enhanced respiratory disease caused by allergic inflammation during the immune process (Fig. 8a). mRNA-1273 demonstrated excellent preventive efficacy and safety, with a 94.1% prevention rate after two 100 μg doses in the phase III clinical trial [27]. However, CVnCoV, developed by CureVac, one of the three leading mRNA technology companies, did not achieve satisfactory results in clinical trials, with an efficacy of only about 47% [220]. CVnCoV employed unmodified nucleosides, which may trigger innate immune activation and lead to mRNA degradation [221]. Moreover, CVnCoV utilized a 12 μg mRNA dose, significantly lower than mRNA-1273 (100 μg) and BNT162b2 (30 μg).

Although mRNA-1273 and BNT162b2 showed favorable overall safety profiles, certain adverse effects were observed in specific populations. The FDA recently approved Moderna’s next-generation COVID-19 vaccine mRNA-1283 for targeted protection of high-risk populations, indicated for all adults aged 65 and older, as well as individuals aged 12–64 with at least one underlying risk factor. mRNA-1283 was administered at a 10 μg dose, but demonstrated higher relative vaccine efficacy compared to mRNA-1273 [222]. Notably, persistent viral mutations may compromise the protective efficacy of existing vaccines, necessitating rapid iterations of mRNA vaccine development.

Compared with the intramuscular injection used for most COVID-19 vaccines, intranasal delivery can deliver the drug directly to the lungs without invasive side effects such as local pain at the injection site. More importantly, intranasal delivery activates mucosal immunity, the body’s first line of immune defense against COVID-19 invasion. Bahl et al. administered LNP-encapsulated mRNA intranasally to hamsters, conferring protection against SARS-CoV-2 infection [223]. The vaccine mitigated severe pulmonary pathology by reducing SARS-CoV-2 lung infection and induced a neutralizing antibody response in serum comparable to that of intramuscular injection controls (Fig. 8b). However, in non-human primates, intranasal delivery of mRNA COVID-19 vaccines failed to enhance mucosal immunity, while adenovirus-based vaccine achieved robust immune response, indicating formulation improvements are necessary for the mucosal delivery of mRNA vaccines [224, 225]. In addition to the active immunity generated by mRNA to activate the immune system, mRNA vaccines can also encode antibodies to neutralize viruses for passive immunity. Tai et al. targeted the mRNA of the monoclonal antibody for SARS-CoV-2 to lungs, bypassing the process of antigen presentation and killing the antigen directly [226].

Building on the success of COVID-19 mRNA vaccines, researchers are continuously optimizing various components of mRNA vaccines to enhance efficacy. ARCT-154 represents the first commercially approved saRNA COVID-19 vaccine, capable of inducing stronger and more durable immune responses at lower doses [23]. At 1 month after vaccination, ARCT-154 (5 μg) and BNT162b2 (30 μg) induced geometric mean titers (GMT) of 2125 and 1624, respectively, for surrogate virus neutralization against Omicron BA.4/5 variants [227]. At 6 months, ARCT-154 maintained significantly higher GMT (1119) compared to BNT162b2 (495). The phase III clinical trial displayed favorable safety profiles for ARCT-154, though long-term risk assessment requires continued monitoring. To boost the immune activation effect of mRNA vaccines, Fan et al. developed a Manganese (Mn)-coordinated mRNA vaccines against SARS-CoV-2 variants [228]. Mn acted as an adjuvant to activate the cGAS-STING pathway and facilitated the maturation of APCs, inducing robust immune reaction in mice. Meanwhile, Mn also increased the transfection efficiency of mRNA by promoting endosomal escape. Currently, commercialized mRNA vaccines utilize LNP as the delivery system, but the clinical application of LNP-mRNA vaccines has been associated with adverse reactions, which partially results from the biotoxicity of LNP, such as anaphylaxis [229], myocarditis [230, 231], and optic neuritis [232]. Mainstream mRNA vaccines including BNT162b2 and mRNA-1273 utilize PEGylated LNPs as the mRNA carrier, which may trigger complement activation and hypersensitivity reactions [233, 234]. Li et al. reported an optimized COVID-19 mRNA vaccine without PEG lipids, stimulating robust immune responses in murine models [235]. Since the decrease of mice weight and cutaneous reactions were not obvious, the safety profile was insured. Apart from the replacement and adjustment of components, Hoffmann et al. introduced a natural infection-mimicking technology by encoding self-assembling enveloped virus-like particles (eVLPs) to improve mRNA vaccines [236]. Compared with conventional mRNA vaccines, the main distinction lies in the extra insertion of the ESCRT- and ALIX-binding regions (EABR). Following mRNA release, protein translation and cell surface expression, the EABR recruits ESCRT proteins to realize the self-assembly of eVLPs budding from cells. Consequently, S proteins were expressed on the surface of host cells and budding eVLPs, which mimicked the natural viral infection process and provided a more effective and long-lasting prevention of SARS-COV-2 infection. The addition of endocytosis-preventing motif (EPM) prolonged the residence time of S-protein at the cytoplasmic membrane, resulting in a more sustainable ESCRT recruitment process (Fig. 8c).

The mRNA vaccine is increasingly recognized as a brilliant tool to prevent epidemics like Zika virus [237], EB virus [238] and herpes simplex virus [239]**.** Scientists in Philadelphia developed a multivalent flu mRNA vaccine, which could provide protection for all known influenza subtypes [240]. Moreover, a malaria mRNA vaccine was performed based on liver-resident memory T cells, showing great potential in a preclinical study [241]. mRNA-1345 is the first FDA-approved mRNA-based respiratory syncytial virus (RSV) vaccine [242]. At a median follow-up of 112 days, the vaccine efficacy against RSV-associated lower respiratory tract disease with at least two signs or symptoms was 83.7%. However, Moderna reported that mRNA-1345 demonstrated only 50% efficacy against RSV at 18-month follow-up. Notably, the vaccine was associated with lower respiratory tract infections in infants, which indicated potential safety concerns requiring further evaluation.

### Cancer Vaccine

Cancer claims millions of lives annually, yet remains inadequately addressed by current therapeutic interventions. Different from preventive epidemic vaccines, most mRNA cancer vaccines are therapeutic, designed to activate the host immune system for targeted tumor cell elimination. Translated from mRNA encoding tumor antigens at ribosomes, proteins are mainly presented by major histocompatibility complex class I (MHC I), which activate CD8^+^ T molecules and induce antitumor cellular immunity.

**Fig. 9:**
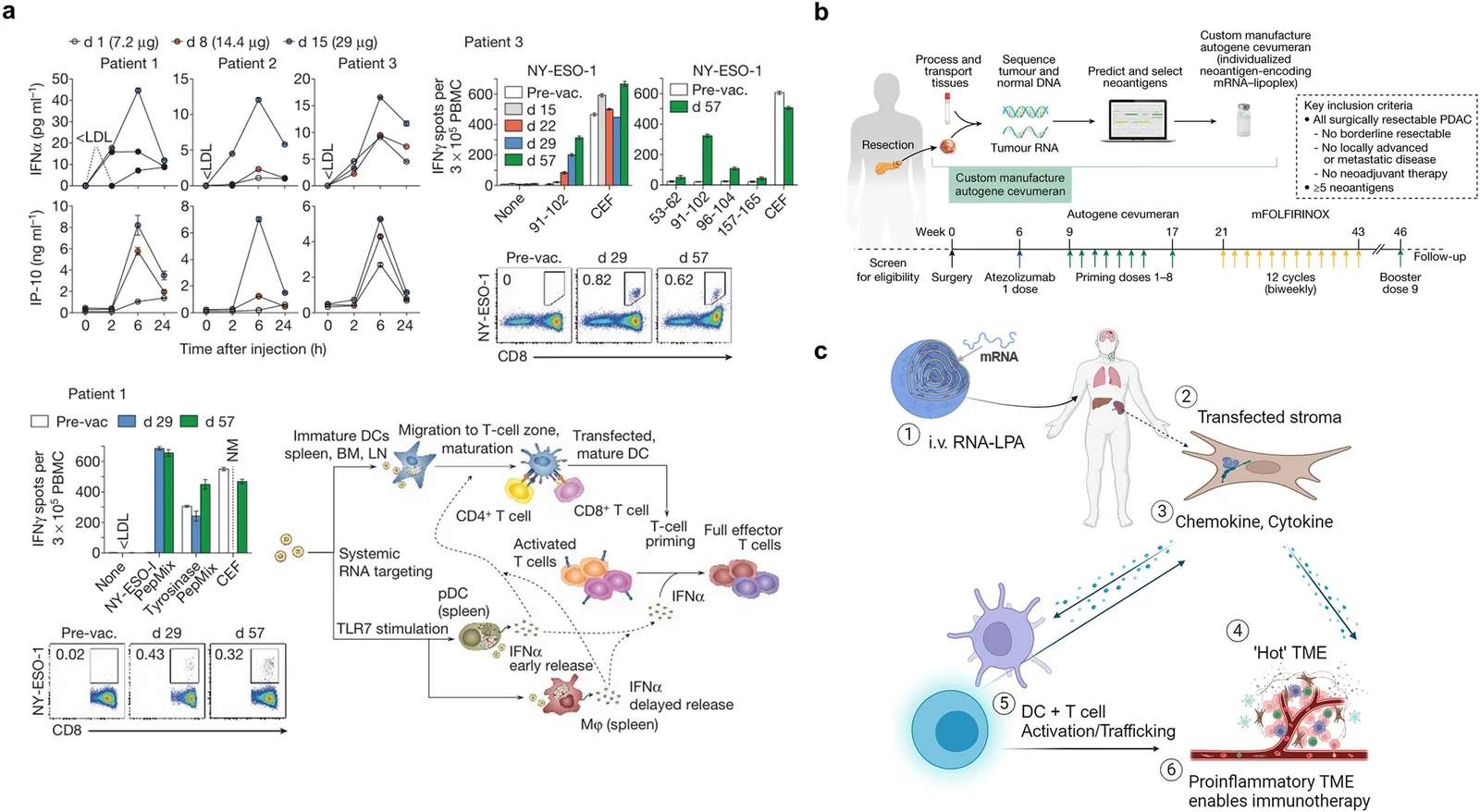
Mechanism and effective treatment outcomes of current cancer immunotherapy. **a** RNA–LPX dose-dependently induced the release of cytokines and lead to the activation of T cells in melanoma patients. Reproduced with permission [247]. Copyright 2016, Springer Nature. **b** Combination therapy of atezolizumab, mFOLFIRINOX and autogene cevumeran (neoantigen vaccine) contributed to a safe and efficient PDCA treatment. Reproduced under the terms of the CC-BY license [30]. Copyright 2023, The Authors, published by Springer Nature. **c** In human glioblastoma patients, LPAs activated innate and adaptive immunity efficiently, reprogramming the tumor microenvironment. Reproduced with permission [254]. Copyright 2024, Elsevier.

The key to mRNA cancer vaccines lies in tumor antigens, including tumor-associated antigens (TAAs) and tumor-specific antigens (TSAs) [243]. TAAs occur in both normal and tumor cells, while the expression often increases when cell carcinogenesis happens, such as HER2 for breast cancer [244], alpha-fetoprotein for primary hepatic carcinoma [245] and PSA for prostatic cancer [246]. Early in 2016, Sahin et al. delivered RNA–liposomes (RNA–LPX) encoding four TAAs (New York oesophageal squamous cell carcinoma 1, melanoma-associated antigen A3, tyrosinase, and transmembrane phosphatase with tensin homology) into dendritic cells as a cancer vaccine [247]. Through the adjustment of charge, the negatively-charged particles targeted the spleen unexpectedly and elicited a powerful immune response in mouse subcutaneous tumor models. RNA-LPX induced DC maturation and stimulated TLR7 in plasmacytoid DCs, which recognized RNA and secreted cytokines, including IFNα, to activate specific immunity. In melanoma patients, the RNA–LPX vaccine elevated systemic IFNα levels, while sensitized and amplified T cells targeting the vaccine antigen (Fig. 9a). Four years later, clinical trial results showed that FixVac, based on RNA–LPX, achieved remarkable outcomes in combination with immune checkpoint blocking therapy, demonstrating its effectiveness as a melanoma immunotherapy [28]. In the 100 μg dose cohort (*n* = 10), 5 patients achieved partial responses, yielding a 50% response rate. The duration of response extended up to 11 months. Furthermore, vaccine-induced PD1 + T cells demonstrated activatability by PD1 antibodies, suggesting potential synergistic antitumor effects. TAA-based mRNA cancer vaccines are required to solve the problem that immune cells regard TAA as their own antigen and thus become tolerant without immune activation [248]. Differently, TSAs are exclusively produced and expressed in cancer rather than healthy tissues, which are regarded as attractive and unique targets [249]. However, the heterogeneity of mutations among patients complicates TSA prediction, making screening a central challenge in cancer vaccine development. mRNA-4157 is an individualized neoantigen therapy targeting up to 34 patient-specific tumor neoantigens, which enhances immune checkpoint inhibitor efficacy by inducing endogenous T-cell responses [29]. mRNA-4157 combined with pembrolizumab significantly reduced the recurrence rate compared to pembrolizumab monotherapy. At the 18-month analysis, the combination therapy group exhibited significantly improved recurrence-free survival (RSF) (79 vs. 62%) and distant metastasis-free survival (92 vs. 77%) compared to monotherapy. Notably, mRNA-4157 induced no grade 4–5 treatment-related adverse events. The compelling efficacy and favorable safety profile enabled mRNA-4157 to become the first mRNA cancer vaccine to advance to phase III clinical trials. For the king of cancer—pancreatic carcinoma, Rojas et al. developed autogene cevumeran, a personalized RNA neoantigen vaccine for pancreatic ductal adenocarcinoma (PDAC) [30]. Current immune checkpoint blockade for PDAC shows little success, while the only cure method—surgery has a high recurrence rate even with multidrug chemotherapy for recurrence delay. Derived from surgically resected tumors, autogene cevumeran comprised a panel containing 20 MHC I and MHC II restricted neoantigens. After surgery, patients received atezolizumab (a PD-L1 antibody), cevumeran and mFOLFIRINOX (a four-agent chemotherapy) in sequence. The combined therapy induced high-intensity neoantigen-specific T cells in half of the vaccinated patients with effective delay of tumor recurrence (Fig. 9b). Recent clinical results based on autogene cevumeran showed that RSF of responders with vaccine-induced T cells prolonged significantly (RFS: median not reached) compared with non-responders without vaccine-induced T cells (RSF: median 13.4 months), indicating effective activation of antigen-specific T cell responses by the vaccine. The vaccine-induced CD8 + T-cell clones exhibited long-term persistence with a projected mean lifespan of 7.7 years, demonstrating durable tumor-responsive capacity [250].

The mRNA therapeutics have been applied to melanoma, prostatic cancer, non-small-cell lung cancer and breast cancer [251]. Besides, Silva et al. reported a mRNA-LNP vaccine designed to treat human papillomavirus infection [252]. For ovarian cancer, Korzun et al. pioneered a mRNA therapeutic addressing metastatic ovarian cancer and cancer-associated cachexia [253]. To inhibit the tumor invasion and metastasis mediated by TGF-β ligand such as ActA, LNPs encapsulating follistatin mRNA were delivered to cancer cell clusters in the peritoneal cavity, reducing ActA levels and alleviating ActA-caused cachexia. For glioblastoma, one of the most aggressive and lethal brain tumors, Elias et al. developed a novel mRNA cancer vaccine based on RNA–lipid particle aggregates (LPAs) [254]. The “onion-like” multi-lamellar structure endowed LPAs with optimized payload packaging, which realized collaborative therapy by delivering multiple RNA drugs jointly. RNA–LPA localized to fibroblastic reticular cells in the spleen and lymph nodes, establishing direct cellular interactions with CD11b+ myeloid cells and APCs. The loop and hairpin secondary structures formed by single-stranded RNA through complementary base pairing enabled RNA–LPA to activate PRR (like RIGI), which induced robust type-I interferon secretion and cytokine/chemokine responses to activate systemic immunity. The vaccine elicited rapid peripheral blood mononuclear cell mobilization to lymphoid organs and tumor sites, which triggered DC activation and CD8+ T cell priming. The infiltrating immune cells mediated immunological conversion from immunologically “cold” to “hot” tumor phenotypes within 48 h. In glioblastoma patients, RNA–LPA similarly elicited robust activation of both innate and adaptive immune responses. Organ function tests of all patients remained stable at acute time points, which demonstrated favorable safety characteristics (Fig. 9c).

### Protein Replacement Therapy

Compared with direct delivery of proteins to cells, mRNA-based protein replacement therapy achieves intracellular protein expression by delivering mRNA encoding a specific protein to target cells, which is equipped with better delivery effects, longer duration of action and relatively lower delivery doses. However, different from the immune-amplifying mechanism of mRNA vaccines in vivo, mRNA protein replacement therapy necessitates higher protein levels to reach the therapeutic threshold, posing significant challenges for mRNA persistence and safety. Table 3 summarizes the dosage ranges of mRNA therapeutics across clinical applications.

**Table 3:** Results of dose escalation in phase I clinical trials

Name	Delivery platform	Application	Clinical trials register identifier	Minimum effective dose	Maximum tolerated dose
BNT162b2	LNP	COVID-19 vaccine	NCT05541861	5 μg	30 μg
mRNA-1273	LNP	COVID-19 vaccine	NCT04283461	25 μg	250 μg
FixVac (BNT111)	Liposome	Melanoma vaccine	NCT02410733	7.2 μg	200 μg
mRNA-4157	LNP	Melanoma vaccine	NCT03313778	0.04 mg	1 mg (No dose-limiting toxicity occurred)
RO7198457	Lipoplex	Pancreatic cancer vaccine	NCT03289962	25 μg	100 μg
CV9103	Protamine	Prostate cancer vaccine	2008-003967-37	256 μg	1280 μg (No dose-limiting toxicity occurred)
RNA-LP	RNA–lipid Particle	Vaccine for newly diagnosed pediatric high-grade gliomas and adult glioblastoma	NCT04573140	Not yet. (Recruiting)	Not yet. (Recruiting)
MEDI1191	LNP	Cytokine therapy	NCT03946800	0.1 μg	12.0 μg (No dose-limiting toxicity occurred)
mRNA-3927	LNP	Protein replacement therapy for propionic acidaemia	NCT04159103	0.3 mg kg^−1^	0.9 mg kg^−1^ (No dose-limiting toxicity occurred)
NTLA-2001	LNP	Gene editing for transthyretin amyloidosis	NCT04601051	0.1 mg/kg	0.3 mg/kg
NTLA-2002	LNP	Gene editing for hereditary angioedema	NCT05120830	25 mg	50 mg (No dose-limiting toxicity occurred)

**Fig. 10:**
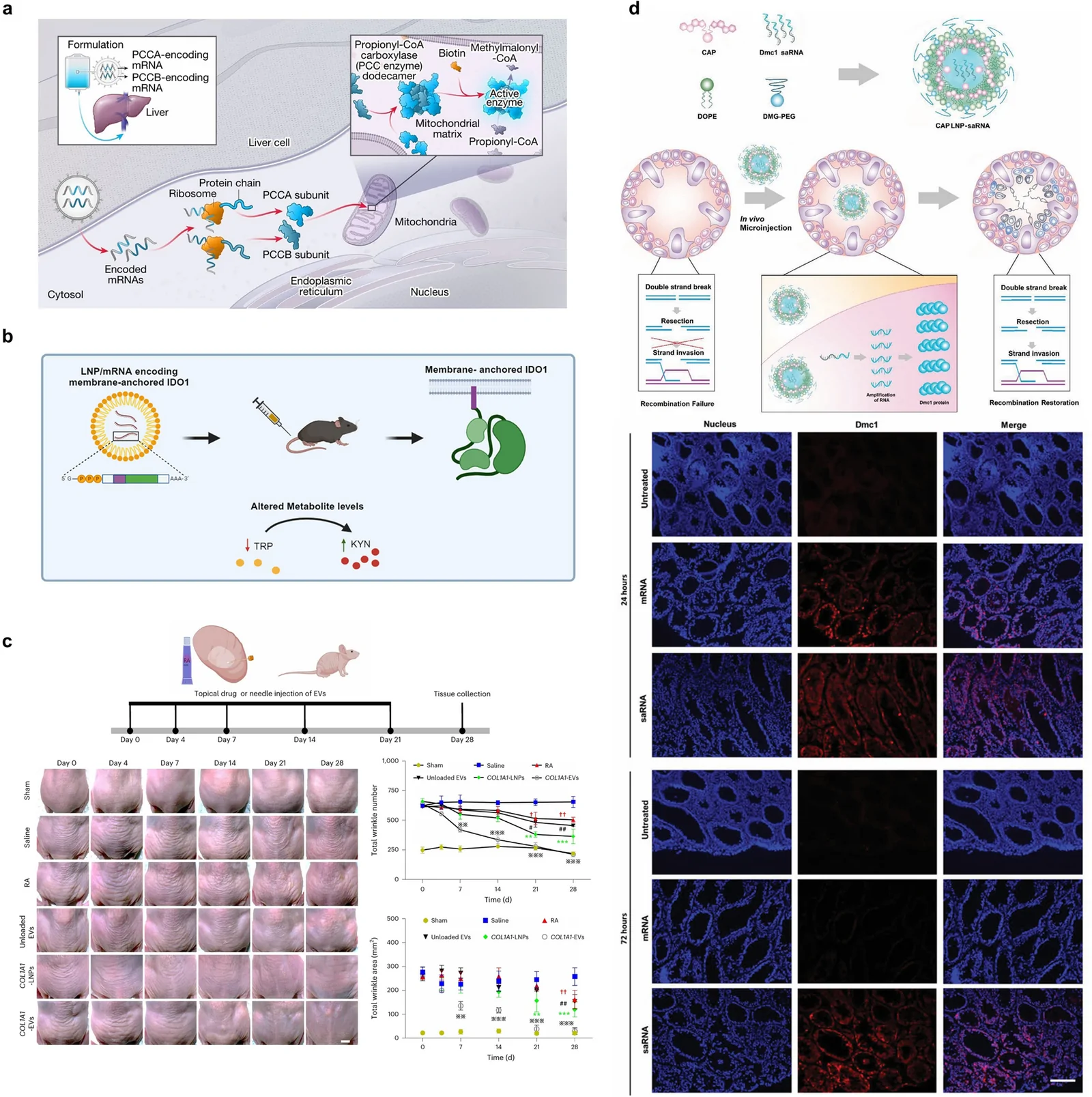
Functional protein expression through mRNA delivery in certain diseases. **a** Dual mRNA encoding PCCA and PCCB was delivered by LNP to express PCC as a treatment of PA. Reproduced under the terms of the CC-BY license [24]. Copyright 2024, The Authors, published by Springer Nature. **b** Modified IDO1 was anchored to the plasma membrane, which increased protein stability and expression. Reproduced with permission [257]. Copyright 2024, Elsevier. **c** EVs loaded with COL1A1-encoding mRNA achieved the decrease of dermal wrinkles in cute photoaging mouse models. Reproduced with permission [258]. Copyright 2023, Springer Nature. **d** CAP LNP with saRNA encoding Dmc1 was microinjected in seminiferous tubules and restored the recombination of chromosome and spermatogenesis in mouse infertility models. Compared with traditional mRNA, saRNA prolonged the protein expression period. Reproduced under the terms of the CC-BY license [261]. Copyright 2023, The Authors, published by John Wiley and Sons.

Protein replacement therapy is especially appropriate for metabolic disorders. For instance, propionic acidemia/aciduria (PA) is a rare and fatal genetic disease. Since the deficiency of propionyl-CoA carboxylase (PCC), a mitochondrial enzyme, leads to the accumulation of harmful metabolites and thus causes PA, Lin et al. delivered two mRNAs encoding α and β subunits of human PCC through LNP to for PCC expression [255]. In a Phase 1/2 clinical trial of mRNA-3927 for PA, the proportion of patients experiencing metabolic decompensation events (a pathognomonic feature of PA) decreased from 50% pretreatment to 12.5% post-treatment. mRNA-3927 exhibited dose-dependent pharmacokinetics. The 0.90 mg/kg cohort demonstrated an extended terminal half-life of approximately 53 h. The reduction of disease-related propionic acid metabolic biomarkers indicated functional restoration PCC activity [24] (Fig. 10a). This represented the first clinical trial utilizing mRNA to express intracellular proteins as a protein replacement therapy in patients with rare diseases. Additionally, Huang et al. successfully delivered mRNA encoding urate oxidase (Uox) for hyperuricemia treatment [256]. Most mammals utilize functional Uox to metabolize insoluble uric acid and maintain physiological serum urate levels, whereas in humans, UOX has evolutionarily pseudogenized through nonsense mutations, representing an atavistic strategy.

Autoimmune diseases represent another promising application for mRNA-based protein replacement therapy. Stein et al. utilized LNPs to deliver mRNA encoding the indoleamine-2,3-dioxygenase 1 (IDO1) variant and inhibited T cell-mediated autoimmunity [257]. IDO1 degrades tryptophan into metabolites of the kynurenine pathway, thereby modulating immune responses. The IDO1 variant incorporated the myristoylation site of Src, anchoring the protein to the inner plasma membrane and prolonging its expression. The delivery of mRNA mediated the overexpression of IDO1 and provided protection in experimental autoimmune encephalomyelitis, rat collagen-induced arthritis, and acute graft-versus-host disease models (Fig. 10b).

In the anti-aging field, You et al. established the EVs encapsulating mRNA encoding extracellular-matrix α1 type-I collagen (COL1A1), which contributed to the decrease of dermal wrinkles [258]. According to a cellular nanoporation method reported before, the authors induced nanochannels by transient electrical pulses to transfect plasmid DNA into cells, followed by transient electrical stimulation to release exosomes loaded with transcribed mRNA [259]. Acute photoaging mouse models were employed to simulate the pathophysiological characters of aging-damaged human skin. Dermal wrinkles were reduced markedly as the translated COL1A1 induced the formation of collagen-protein grafts, demonstrating its efficacy in the recovery of photoaged dermis and the clinical potential of mRNA delivery for anti-aging and collagen replacement. Furthermore, the application of a microneedle array extended the collagen–protein replacement period and enabled more uniform EV delivery in tissues (Fig. 10c). Since EV secretion is a natural property of cells, Nawaz et al. aimed to observe the extended mRNA transportation via EVs among cells [260]. The mRNA encoding VEGF-A, an angiogenic molecule, was delivered by LNP to targeted ischemic tissues for production of new blood vessels. After the successful delivery of VEGF‐A mRNA, LNP was taken into cells through endocytosis. EVs were then secreted out and the copy of VEGF‐A mRNA could be detected in the supernatants where large amount of EVs accumulated, displaying an extended function to distribute therapeutic mRNA among cells.

The advantages of saRNA over mRNA in protein replacement therapy are significant, including higher protein expression levels and prolonged half-life. Du et al. developed a range of cholesterol‐amino‐phosphate (CAP) LNPs to deliver mRNA or saRNA encoding DNA Meiotic Recombinase 1 (Dmc1) to spermatocytes and thus treat male infertility caused by the Dmc1 gene mutation [261]. The integrated CAP LNPs were designed to promote phase transformation, endosome escape and RNA release, which were microinjected into seminiferous tubules in Dmc1-gene knockout mice. Dmc1 protein expressed by exogenous saRNA restored the recombination of chromosome and spermatogenesis with longer protein expression than mRNA group. The application of saRNA in infertile mice sets a precedent in treating genetic diseases other than immunotherapy, showing potential as an effective pathway to cure male infertility (Fig. 10d).

### Cytokine Therapy

Cytokines are considered as either cancer targets or treating means during the past decades [262]. The delivery of cytokines can reverse the tumor immunosuppressive microenvironment on one hand and enhance antitumor immunity on the other. However, the pleiotropic characteristic of cytokines and the widespread presence of cytokine receptors in various tissues lead to systemic toxicity if cytokines are delivered systemically, so in situ delivery of cytokines to tumors is necessary. Cytokines leak into the circulation quickly after direct intratumoral injection, leading to a short retention time at the tumor sites, while mRNA is able to achieve long-lasting cytokine expression. IL-12 exhibits various biological activities, including enhancing the activity of NK cells and cytotoxic T cells, stimulating the proliferation of activated NK cells or T cells, and inducing the release of IFN-γ [263], so it is a popular cytokine for mRNA encoding. Luheshi et al. achieved localized production of IL-12 at the tumor sites through intratumoral delivery of IL-12 mRNA, which induced IFN-γ expression and promoted TH1 transformation of the tumor microenvironment [31]. MEDI1191 derived from this research has already entered Phase 1 clinical trial, displaying favorable safety and antitumor activity. Hunter et al. extended the application of IL-12 mRNA from cancer treatment to vaccine optimization, regarding IL-12 as a vaccine adjuvant [264]. IL-12 enhanced mRNA vaccine-induced CD8 T cell expansion and promoted memory T cell differentiation, providing a solution to the decline in antibody titers observed with mRNA vaccines.

**Fig. 11:**
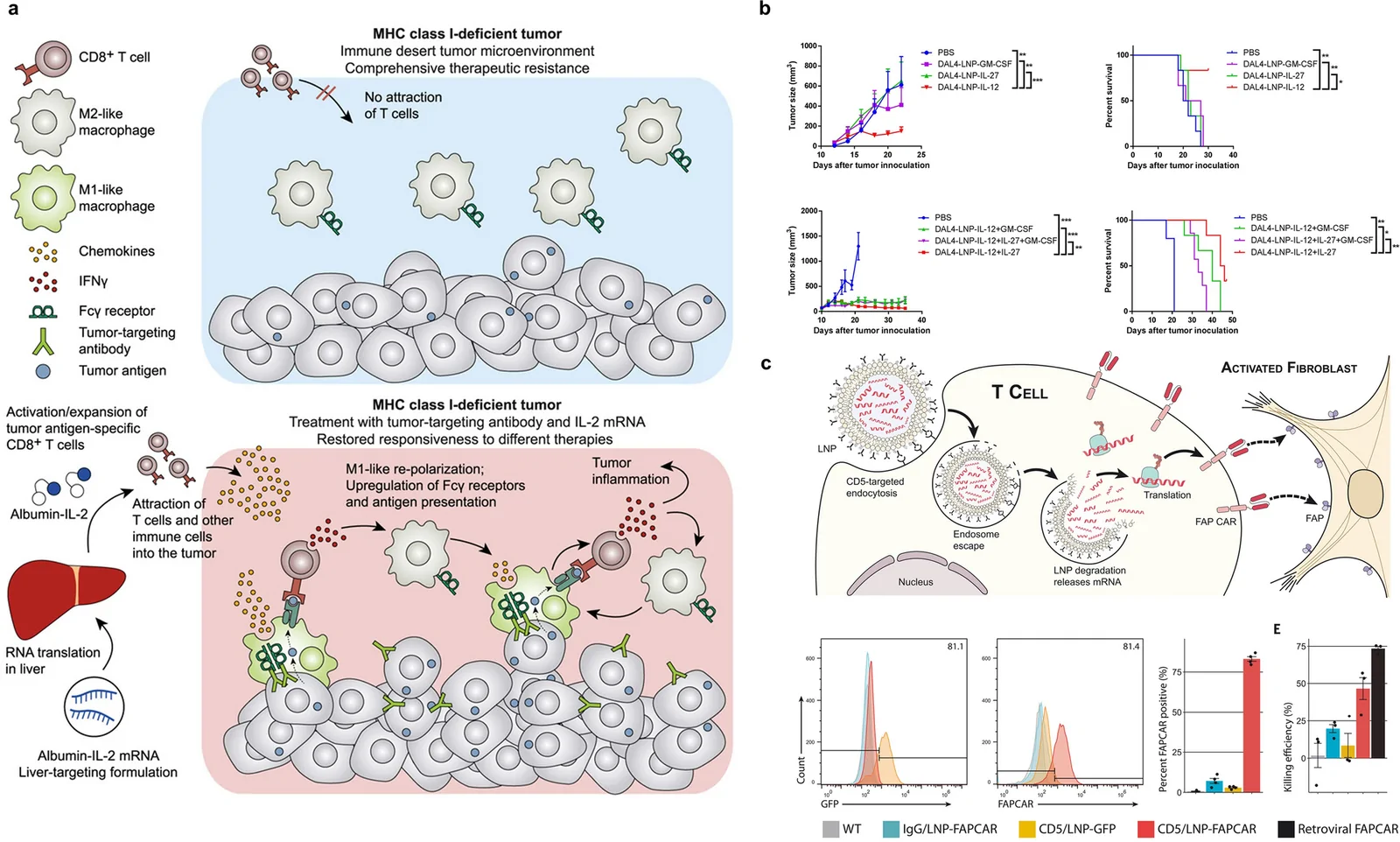
mRNA-based cell therapy and cytokine therapy. **a** Albimin-IL-2 mRNA facilitated the M1 polarization of macrophages and enhanced antigen presentation by CD8^+^ T cells, thereby restoring immune cell infiltration and ameliorating the tumor microenvironment. Reproduced with permission [266]. Copyright 2022, Elsevier. **b** mRNA encoding IL-12 and IL-27 displayed strongest tumor inhibitive effect compared with mRNA encoding one cytokine. Reproduced with permission [268]. Copyright 2022, Elsevier. **c** CD5-targeted LNP-mRNA was delivered to T cells in heart failure mouse models and enabled in vivo generation of CAR-T. FAP-targeted CAR-T achieved a transient antifibrotic effect without long-term cytotoxicity. Reproduced with permission [141]. Copyright 2022, The American Association for the Advancement of Science.

IL-2 plays a crucial role in the expansion and differentiation of immune cells, which is another cytokine commonly used in cytokine therapy [265]. To prolong the short serum half-life of IL-2, Sahin et al. designed a lipid-based nanoparticle complex encoding albumin-fused IL-2, enabling specific delivery to the liver [266]. In a tumor model of MHC I-deficient mice, IL-2 transformed tumor-associated macrophages into M1 macrophages, which promoted cross-presentation of antigens to CD8^+^ T cells and thus restored immune cell infiltration (Fig. 11a). IL-2 promotes immune responses at high concentrations, while it preferentially activates Tregs at lower concentrations [267]. mRNA-6231 encoded a modified human interleukin 2 mutein fused to human serum albumin (HSA‐IL2m), which holds significant potential for autoimmune diseases [32].

Compared with delivering mRNA encoding one single cytokine, the co-delivery of mRNA encoding multiple cytokines can elicit a more extensive tumor immune response. Liu et al. reported an LNP-loaded mRNA cancer vaccine encoding IL-12 and IL-27 [268]. The intratumoral delivery of mRNA encoding IL-12/IL-27 not only restrained the growth and deterioration of the tumor, but also resulted in the strong infiltration of immune effector cells in the tumor, which reversed the tumor immunosuppressive microenvironment and created favorable conditions for cytotoxic T lymphocytes (Fig. 11b). Other combinations included a mixture of mRNAs encoding IL-23, IL-36, and OX40L, as well as mRNAs encoding GM-CSF, IFNα, IL-15–sushi and scIL-12 [269, 270].

### Cell Therapy

CAR-T therapy represents a revolutionary cell therapy and has achieved significant breakthroughs in hematologic malignancies (targeting CD19), which collects T cells from the peripheral blood of patients or donors and modified the cells genetically ex vivo for the expression of CARs [271]. CAR contains an antigen-recognition domain, a CD3ζ activation domain and a costimulatory domain, which can recognize specific surface antigens on tumor cells independent of antigen presentation [272]. mRNA encoding CD19-targeted CAR-T has already entered the clinical trial [33, 34]. mRNA-based CAR-T technology often employs electroporation to transfect mRNA into T cells ex vivo for the generation of CAR-T cells, which demands high costs and extensive preparation time [273]. Epstein et al. developed an mRNA-based CART cell heart injury therapy that could produce CAR-T cells in vivo [141]. CD5-targeted LNP delivered mRNA to T cells without the influence of T cell effector function. mRNA encoded a CAR that targeted at fibroblast activating protein (FAP), a marker for activated fibroblasts, which allowed T cells to eliminate activated fibroblasts in the heart to treat heart failure caused by fibrosis. The short half-life of mRNA meant that this CAR-T cell production was transient, thus avoiding the toxic effects of long-term inhibition on fibroblasts (Fig. 11c). Three years later, the authors applied this mRNA-based in vivo CAR-T technology to B cell-mediated autoimmune diseases [274]. To prevent adverse effects and cytokine release syndrome from CD4 + T cells, the LNP was modified with CD8 antibodies for specific targeting of CD8 + T cells. The mRNA encoding anti-CD19 CAR (or anti-CD20 CAR in cynomolgus monkeys) enabled the generated CAR-T cells to eliminate pathogenic B cells precisely. The reconstituted B cells after depletion were predominantly of a naïve phenotype (85%), achieving an “immune reset”.

In the CAR-NK cell therapy, CD19 is also one of the popular targeted antigens. NK cells are enabled to target and kill cancerous B cells expressing CD19 after mRNA electroporation [275]. CD20 (a B cell differentiation antigen), natural killer group 2 member D ligand (NKG2DL) and B cell maturation antigen have also been employed as targets for CAR-NK [276].

Macrophages can infiltrate solid tumor tissues and overcome target antigen heterogeneity, so CAR-M therapy is regarded as a promising approach for solid tumors, which aims to enhance immune responses by converting M2 macrophages into the M1 phenotype [277]. Liu et al. achieved in situ construction of CAR-M cells by modifying LNPs with phosphatidylserine for macrophage-specific uptake [278]. Meanwhile, the authors analyzed various CAR constructs containing different intracellular domains (Phagocytosis: CD3ζ and Dectin1; proinflammatory: CD40 and TLR4; and possible effector: CD46 and CFS2R) to achieve optimal CAR-M efficacy.

### Gene Editing

The field of gene editing develops rapidly in recent years, while Cas protein delivery still lacks an economic and efficient system. Therefore, nucleic acid delivery shows great potential for application. Compared with the unavoidable risk of exogenous gene insertion of DNA, mRNA can express proteins required for gene editing safely and directly in cells, which is a promising alterative.

**Fig. 12:**
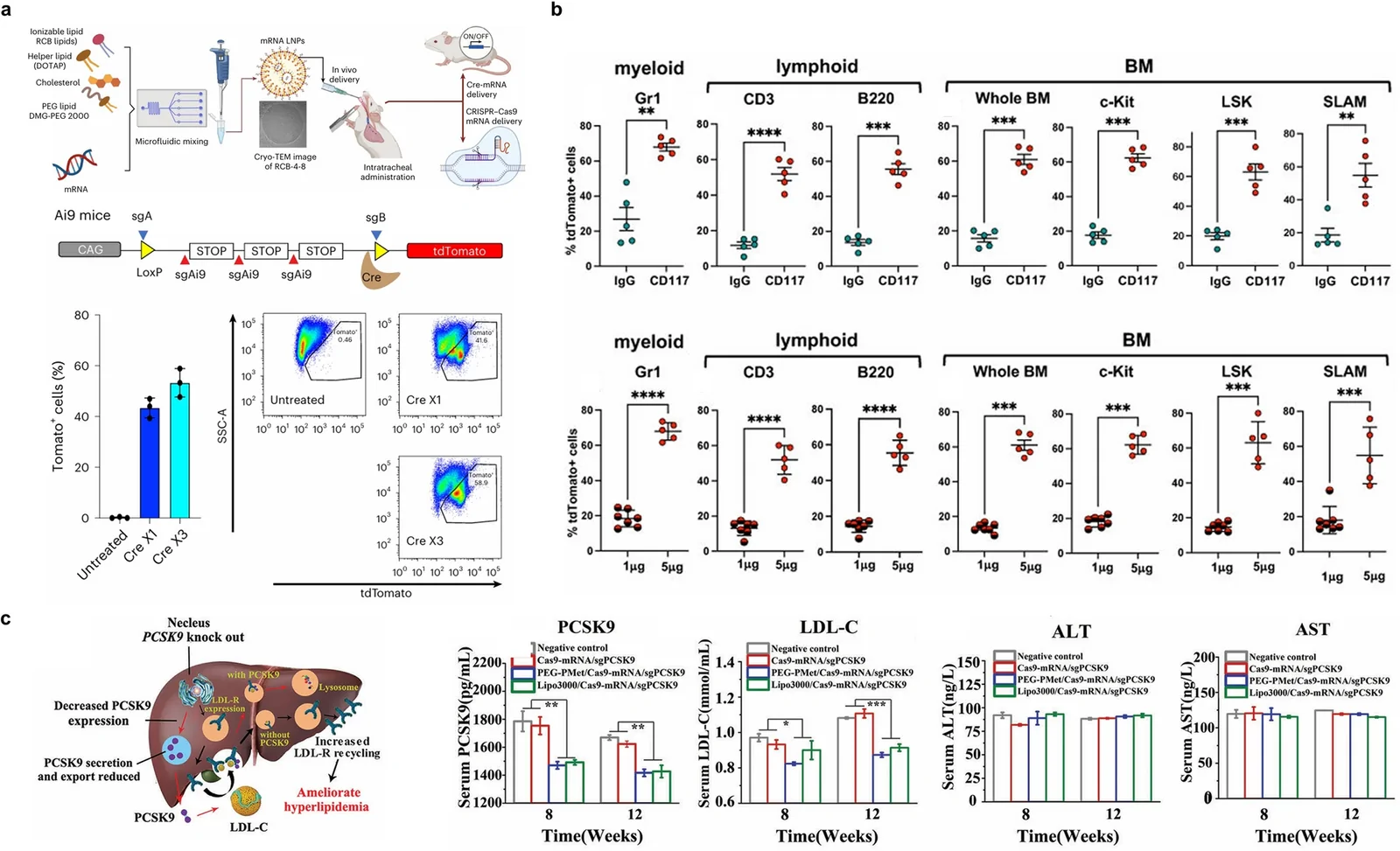
mRNA-based technology in the application of gene editing. **a** Cre translated from exogenous mRNA recognized the Loxp sequences, which deleted STOP cassettes and activated the tdTomato reporter. Reproduced with permission [279]. Copyright 2023, Springer Nature. **b** Following CD117/LNP-Cre injection in reporter murine models, significant tdTomato marking was observed in bone marrow subsets and peripheral blood myeloid and lymphoid cells, demonstrating long-term editing. Reproduced with permission [280]. Copyright 2023, The American Association for the Advancement of Science. **c** Knockout of the gene reduced the level of LDL in serum, ameliorating hyperlipidemia without inducing significant hepatic injury. Reproduced under the terms of the CC-BY license [286]. Copyright 2023, The Authors, published by John Wiley and Sons.

The Cyclization Recombination Enzyme—locus of X-over P1 (Cre-Loxp) system is an important gene editing platform. Cre is a recombinase that specifically recognizes Loxp sequences and induces recombination of DNA located between these two Loxp sequences, which can be encoded by mRNA and thus function after translation in vivo. Li et al. designed a three-component reaction system and built a combinatorial library of ionizable lipids to construct nanoparticles for pulmonary mRNA delivery, which consisted of ricinoleic acrylate linker, aliphatic alcohols (lipid tails) and amine headgroups [279]. The ionizable lipids in the library contained abundant ester and carbonate groups, endowing them with excellent biodegradability suitable for repeated administration. Cre mRNA was delivered by LNP to airway epithelium in the Lox-3xSTOP-Lox(LSL)-tdTomato reporter mice (Ai9), and translated into Cre recombinase, which recognized the two LoxP sequences and removed the LSL cassette, resulting in downstream tdTomato transcription. After Ai9 mice were treated with one (LNP-Cre × 1) or three (LNP-Cre × 3) doses, obvious red fluorescence of tdTomato signal was detected in lung by flow cytometry, indicating the effect of gene editing was dose-dependent. Compared with DNA-Cas9 delivery system, the relatively short existing time of mRNA avoids the influence caused by off-target effect, while LNP can be repeatedly dosed and thus solve the difficulty in achieving therapeutic editing levels, which is a significant advantage over viral vectors (Fig. 12a). In the treatment of blood diseases, Laura et al. modified LNP with CD-117 antibodies and developed a gene editing platform targeting hematopoietic stem cells (HSCs), where CD-117 are overexpressed [280]. In murine models, intravenous injection of CD117/LNP-Cre achieved gene editing within 24 h, with sustained expression persisting over the subsequent 4 months. The long-term HSC editing rate reached 55%, achieving efficient and durable gene editing of HSCs. Compared with current gene therapies predicated on chemotherapy and stem cell transplants, this study offers a once-for-all treatment for blood disorders such as sickle cell disease and beta-thalassemia (Fig. 12b).

CRISPR-Cas9, consisting of Cas9 and sgRNA, is another efficient genome editing tool available [281]. Cas9 mRNA and sgRNA are complexed with vectors and delivered to cells to realize gene editing in treatment of various diseases, such as hemophilia A and B therapy [282], cervical cancer [178], acute myeloid leukemia [283] and liver diseases [284]. An early report on the treatment of Transthyretin amyloidosis by LNP encapsulating Cas9 mRNA and transthyretin (TTR)-targeted sgRNA has already entered the third clinical phase (NTLA-2001), which was the first in vivo CRISPR gene editing therapy approved for advanced clinical development [21]. After intravenous administration, the LNPs in NTLA-2001 bound to plasma apolipoprotein E and were endocytosed by hepatocytes via the LDL receptors, enabling efficient liver-targeted delivery. Since the liver produces the majority of TTR, this strategy effectively reduced systemic toxicity. After cell entrance, expressed Cas9 protein edited DNA at TTR gene sequence coordinating with sgRNA and blocked the misfolded TTR production. On day 28 after NTLA-2001 treatment, the 0.1 and 0.3 mg kg^−1^ dose groups exhibited mean TTR reductions of 52% and 87%, respectively, demonstrating a dose-dependent effect. Similar with NTLA-2001, NTLA-2002 is also based on CRISPR-Cas9 system for the treatment of hereditary angioedema [35]. Inhibitors of the plasma kallikrein activity is considered suitable for long-term prophylaxis but lifelong administration is inevitable. NTLA-2002 can permanently edit the gene responsible for the production of plasma prekallikrein (KLKB1). LNP delivered mRNA encoding the Cas9 endonuclease and sgRNA targeting KLKB1 to the liver, which disrupted the production of plasma prekallikrein and thus reduced the total plasma kallikrein protein level. In the 25, 50, and 75 mg groups, the mean percentage changes in total plasma kallikrein protein levels were − 67, − 84, and − 95%, respectively. Plasma kallikrein activity also showed dose-dependent reduction, closely correlating with the decrease in total plasma kallikrein protein levels. Recently, mRNA gene editing systems have also been employed for inherited retinal diseases, with successful gene editing in retinal epithelium and Müller glia [285]. Additionally, Zhao et al. developed a treatment for hyperlipidemia amelioration by disrupting PCSK9 gene [286]. The ester bonds introduced into the carrier were hydrolyzed by hepatic carboxylesterase 1, enabling controlled mRNA release in the liver. The expression of PCSK9 was decreased and LDL receptors were upregulated, increasing the uptake of LDL to liver. Compared with non-treatment group, serum total cholesterol decreased by 20%, with no significant changes in alanine aminotransferase or aspartate aminotransferase levels, demonstrating favorable biosafety (Fig. 12c).

Mutated from Cas9, dCas9 is deprived of the endonuclease activity so it is applied in studies of endogenous gene expression regulation [287]. Beyersdorf et al. reported that the successful and lasting activation of transcriptional and epigenetic gene activation through delivery of LNP with mRNA and sgRNA [288]. The mRNA encoded fusion proteins of dCas9 and a transcription activating domain, which activated the B4galnt2 gene to induce up to 1000-fold mRNA overexpression. The cell activation rate reached 90%, while the kinetics of gene activation showed a high copy level lasting for 9 days. The mRNA encoding AcrIIA4 protein was to prevent the combination of dCas9 activator and genomic DNA, which inhibited the sustained gene activation to improve the safety profile. Besides, the erythropoietin gene demonstrated the feasibility of the approach in other target genes. Splenomegaly indicated the increase of blood circulation because of the rise of erythrocytes, proving the success of gene activation. Compared with protein replacement therapy, this dCas9 mRNA delivery system displays a broader pharmacokinetic curve of therapeutic protein with one single dose.

## Challenges and Potential Solutions

With the deepening of research on mRNA structural optimization and delivery strategies, mRNA therapeutics are maturing, but the clinical applications still face significant challenges.

A pressing issue is the inability of protein replacement therapy to provide sustained protection, raising the critical question of how to prolong mRNA functionality. While existing structure optimization tools, such as LinearDesign, have significantly extended the half-life of mRNA, maintaining protein expression for more than a week remains difficult due to the single-stranded nature of mRNA. In addition, the instability of mRNA is compounded by its immunogenic nature, as exogenous mRNA is recognized as a pathogen-associated molecular pattern by TLRs in vivo. Nucleoside modification to reduce mRNA immunogenicity is an essential and crucial approach. However, certain modifications, such as N1-methylpseudouridylation, have been shown to induce ribosomal frameshifting, leading to the production of unintended proteins [289]. This unpredictability is a potential safety concern for mRNA-based therapeutic strategies. To overcome limitations of linear mRNA, alternative mRNA variants such as circRNA and saRNA have been developed to achieve sustained expression. circRNA has a 2.5-fold longer half-life than linear mRNA, though challenges remain in designing an effective IRES sequence [109]. saRNA, on the other hand, requires 30–1000 times less dosage to achieve the same protein expression levels as linear mRNA [85], making it a promising option for protein replacement therapies that require large amounts of protein with long-term expression. Importantly, the inclusion of viral-derived sequences in saRNA confers self-adjuvant properties, prompting a reconsideration of its relationship with immune response intensity.

Apart from the optimization of mRNA structure, delivery vehicles represent another major determining factor for mRNA therapeutics. The toxicity of polymeric vehicles limits their clinical translation, while the delivery efficiency of peptides/proteins, exosomes and polyphenols remain limited. Contributing to the remarkable success of mRNA vaccines, LNP represents the most widely used and mature mRNA delivery vehicle. Commercialized mRNA vaccines such as mRNA-1273 and BNT162b2 have been associated with unpredictable side effects that are likely related to LNP components. Optimization of LNP composition, such as screening lipid types and ratios, can reduce the immunogenicity and toxicity of delivery systems. As an alternative to PEGylated lipids, the PCB–lipid addresses the issues of non-degradability and repeated administration, undoubtedly representing an exemplary case. The design of hybrid nanoparticles is a feasible compensatory approach combining the advantages of different materials. Furthermore, targeted delivery is a feasible pathway to enhance delivery efficiency and reduce off-target toxicity. Alterations of physicochemical properties through LNP component adjustment offers a promising solution to improve organ-targeted delivery. Moreover, modification with specific targeting moieties endows delivery vehicles with cell-specific targeting capability, achieving more precise mRNA delivery.

The success of COVID-19 vaccines has accelerated the spread of mRNA technology across various medical fields. Personalized cancer vaccines represent a promising area, while neoantigen screening is a key challenge. Although whole-exome sequencing and bioinformatics tools can identify tumor mutations, high false-positive and false-negative rates persist in neoantigen prediction [290]. Tumor heterogeneity and unpredictable mutations, such as single nucleotide polymorphisms, often lead to the failure of neoantigen-based cancer vaccines. Moreover, a substantial proportion of neoantigens in vaccines fail to elicit T cell responses, necessitating additional strategies to enhance APC functionality and T cell priming in lymph nodes. Costimulatory receptor agonists and TLR agonists represent potentially effective strategies. The efficacy of cancer vaccines can also be significantly enhanced by modulating cytokines to improve the tumor immunosuppressive microenvironment.

Personalized cancer vaccines may also provide insights into tailoring epidemic vaccines for different populations. Variations in immune function across populations indicate that mRNA-based products, such as the COVID-19 vaccines, are not equally suitable for all individuals. Individuals with underlying diseases often experience more severe side effects such as fatigue, inflammation, and exacerbation of underlying diseases, which can be fatal in some cases. Older adults are more susceptible to complications such as myocardial infarction and Guillain-Barré syndrome, while younger individuals are more susceptible to myocarditis and anaphylaxis [291, 292]. Therefore, the development of mRNA vaccines tailored to specific groups, such as those with or without underlying health conditions or for different age groups, may be a viable strategy to reduce adverse effects and improve vaccine efficacy.

In the manufacture of mRNA-based therapeutics, the safety and purity of raw materials are critical factors that directly affect the quality of the final product. The selection of raw materials, such as plasmid DNA (pDNA) serving as the template for mRNA production, must comply with pharmaceutical regulatory guidelines. However, due to intense market competition, it is particularly challenging for new companies to obtain high-quality pDNA, which has a significant impact on subsequent production processes. In the purification of mRNA, rigorous removal of impurities such as dsRNA through high-performance liquid chromatography can significantly reduce the immunogenicity of the final products, since dsRNA is a potent innate immune activator. In addition, the inherent instability of mRNA is a major barrier to its storage. For example, mRNA-1273 requires storage at − 20 °C, while BNT162b2 must be transported and stored at temperatures ranging from − 80 to − 60 °C [293]. Once thawed, BNT162b2 is only functional for 2 h at room temperature. The stringent storage conditions significantly increase costs and pose significant barriers to the widespread use of mRNA vaccines in underdeveloped regions. Lyophilized mRNA-LNP vaccines have been proposed as a potential solution [294, 295], but whether the efficacy is compromised remains to be verified. As mRNA technology continues to advance rapidly, the establishment of standardized regulations for production, storage, and institutional practices will be essential for the maturation and wider adoption of mRNA-based therapeutics. Regulatory agencies worldwide should enhance communication and collaboration to promote the international harmonization of regulatory standards for mRNA therapeutics. Given that mRNA remains an emerging technology, the long-term effects of mRNA therapeutics on human cell physiology remain unknown. As mRNA vaccines such as mRNA-1273 have been approved by regulatory agencies such as the FDA at an unprecedented pace, some steps in the review process may have been overlooked. Therefore, it is critical to conduct long-term follow-up studies to assess potential disease risks and changes in disease spectra especially among specific populations.

## Future Perspectives

As an emerging therapeutic approach with immense potential, mRNA holds broad development prospects for the future. Compared with traditional manual design, LinearDesign and circDesign demonstrate unparalleled advantages and efficiency in the design and optimization of mRNA structures, indicating that rapidly advancing artificial intelligence (AI) will play an increasingly indispensable role in the field of mRNA. In delivery vehicle optimization, AI offers a crucial supplementary approach to current experimental screening. Regarding LNP, the most advanced mRNA delivery carrier, researches have utilized AI deep learning to predict key properties of ionizable lipids, which accelerates the screening process, optimizes lipid performance and reduces the cost associated with traditional experimental screening significantly [296]. In addition to efficient optimization of one individual component, adjusting the proportions of LNP components and optimizing physicochemical properties can realize the preparation of optimal LNPs and endow LNP with specific functionalities, such as targeted delivery. When it comes to mRNA application, AI can also predict the affinity between antigens of mRNA infectious disease vaccines and immune receptors, determining whether they can elicit sufficient immune responses to generate immunological memory [297]. In the development of mRNA cancer vaccine, AlphaFold can accurately predict protein structures, which enables the tailored design of mRNA sequences encoding neoantigens [298]. Therefore, AI is poised to progressively transform the entire process of lifecycle of mRNA therapeutic development. Notably, AI-driven design also faces numerous challenges. High-quality and large-scale datasets are essential for training AI models, while data acquisition remains a significant obstacle, which entails substantial computational resources and prolonged training periods concomitantly. The gap between in silico models and real-world implementation may exist, given the inherent challenges in modeling intricate biological networks. While models demonstrate robust performance on specific training datasets, their efficacy may significantly decline in real-world applications due to distributional shifts between training and deployment environments. For instance, structurally optimized mRNA constructs may trigger unintended immune responses [84]. Moreover, current models exhibit limited generalizability, raising concerns about whether training on specific population cohorts and datasets can ensure robust extrapolation to broader applications. Population diversity contributes to aberrant immunogenicity and efficacy profiles of mRNA therapeutics in specific subpopulations. Developing more efficient AI models presents a viable strategy to address current bottlenecks. Establishing privacy-preserving data-sharing platforms will enhance training data diversity for AI models. Integrating multi-omics data (such as proteomics, immunopeptidomics) will further improve predictive capabilities.

mRNA delivery technologies will continue to advance through iterative innovation. LNPs remain the predominant delivery vehicles in clinical-stage development, with ongoing optimizations targeting their immunogenicity and toxicity profiles. Meanwhile, endogenous carriers demonstrate inherent advantages in biocompatibility. Natural occurring human proteins like Peg10 can elicit fewer immune responses and side effects, which ensure the safety and therapeutic effect of repeated administrations [157]. Exploring strategies to enhance the delivery efficiency of Peg10 like the modification with endogenous cell-penetrating peptides may offer new alternatives for mRNA delivery.

The high programmability of mRNA and the functional diversity of proteins will extend and deepen its applications to a wide range of areas. Active and passive immunity remain core mechanisms of mRNA-based therapeutics, providing preventive and therapeutic strategies for a broader spectrum of diseases. mRNA vaccines targeting bacteria have been reported, showing promising efficacy in the prevention of tuberculosis and Clostridioides difficile infection [26, 299]. Further development of therapeutic bacterial vaccines will undoubtedly provide an essential complementary treatment for the increasingly severe issue of bacterial drug resistance. Additionally, mRNA vaccines encoding parasitic antigens, such as malaria antigens, offer potential interventions for a wide range of parasitic infections [300]. mRNA encoding disease-related autoantigens have been shown to stimulate antigen-specific regulatory T cells and mediate immune tolerance [301]. mRNA will enable targeted expression of functional proteins across expanding therapeutic domains. In regenerative medicine, in addition to delivering mRNA encoding regeneration-related factors directly to induce tissue regeneration [302], reprogramming mature somatic cells into pluripotent stem cells through in vitro mRNA transfection may be another attractive strategy.

## Conclusions

Decades of research have culminated in the rapid development of mRNA-based therapeutics. The success of mRNA COVID-19 vaccines has highlighted the significant advantages of this approach, including promising preventive and therapeutic efficacy, safety with respect to genetic mutations, relatively low production costs and rapid manufacturing. These attributes have captured the attention of the scientific community and spurred extensive research into the fundamental components of mRNA.

While the path forward for mRNA-based therapeutics may not be straightforward with challenges remaining, the immense potential of this technology is undeniable. Undoubtedly, mRNA-based therapeutics are poised to revolutionize current drug development paradigms. This transformative technology not only enables pharmaceutical manufacturers to rapidly adapt production pipelines to emergent needs, but also accelerates the industry transition toward personalized and precision medicine, which delivers more comprehensive and timely responses to medical demands and provides patients with significantly improved therapeutic precision. Real-time medicine, which integrates rapid response and precision medicine, will be enabled by mRNA therapeutics. Further breakthroughs in mRNA structural modifications and delivery systems are on the horizon, paving the way for more mature and innovative mRNA-based therapeutic strategies that can benefit humanity profoundly.
